# Coevolution underlies GPCR-G protein selectivity and functionality

**DOI:** 10.1038/s41598-021-87251-6

**Published:** 2021-04-12

**Authors:** Min Jae Seo, Joongyu Heo, Kyunghui Kim, Ka Young Chung, Wookyung Yu

**Affiliations:** 1 Department of Brain and Cognitive Sciences, DGIST, 333 Techno jungang-daero, Daegu, 42988 Republic of Korea; 2 Department of Undergraduate Studies, DGIST, 333 Techno jungang-daero, Daegu, 42988 Republic of Korea; 3 Core Protein Resources Center, DGIST, 333 Techno jungang-daero, Daegu, 42988 Republic of Korea; 4 School of Pharmacy, Sungkyunkwan University, 2066 Seoburo, Jangan-gu, Suwon, 16419 Republic of Korea

**Keywords:** Computational biophysics, Protein sequence analyses, G protein-coupled receptors

## Abstract

G protein-coupled receptors (GPCRs) regulate diverse physiological events, which makes them as the major targets for many approved drugs. G proteins are downstream molecules that receive signals from GPCRs and trigger cell responses. The GPCR-G protein selectivity mechanism on how they properly and timely interact is still unclear. Here, we analyzed model GPCRs (i.e. HTR, DAR) and Gα proteins with a coevolutionary tool, statistical coupling analysis. The results suggested that 5-hydroxytryptamine receptors and dopamine receptors have common conserved and coevolved residues. The Gα protein also have conserved and coevolved residues. These coevolved residues were implicated in the molecular functions of the analyzed proteins. We also found specific coevolving pairs related to the selectivity between GPCR and G protein were identified. We propose that these results would contribute to better understandings of not only the functional residues of GPCRs and Gα proteins but also GPCR-G protein selectivity mechanisms.

## Introduction

G protein-coupled receptors (GPCRs) are one of the most important signal transduction systems to transmit extracellular signals (e.g., light, odorants, and hormones) into cells. GPCRs regulate critical physiological functions including sense recognition, neural transmission, and hormonal responses^1^. Since they play important roles in human physiology and pathology^2^, GPCRs have been extensively studied over the past decades. However, a number of questions regarding GPCR systems still remain to be addressed, and moreover new phenomena have been identified day by day in recent years. Current active research into GPCRs would help to understand the fundamentals of cell signaling and would be useful for GPCR-targeted drug developments^2–4^.

**Figure 1:**
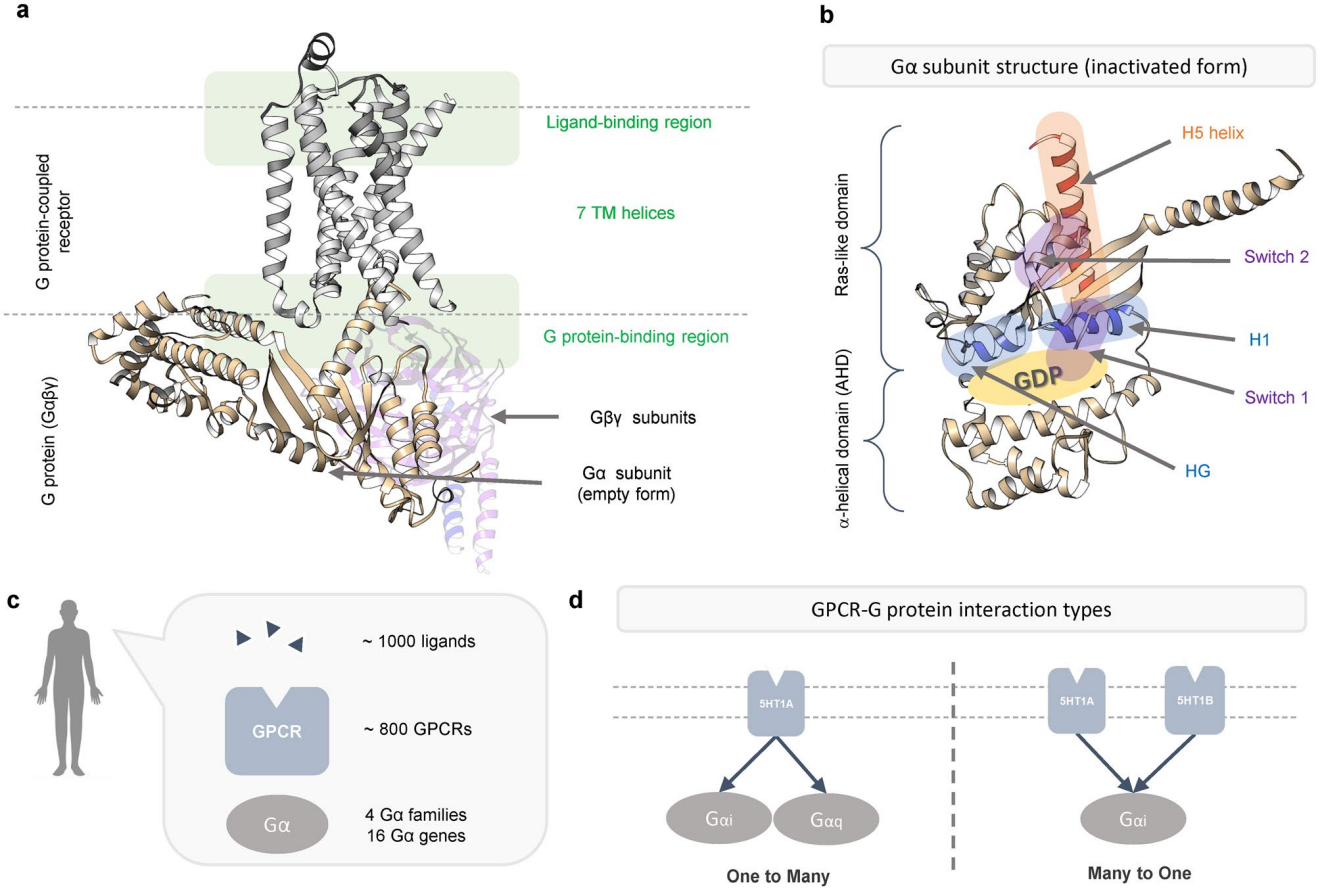
Structure and selectivity of GPCR and G protein. (**a**) Structure of GPCR-G protein complex. This structure is obtained from GPCRdb (modeling structure of 5HT2B-Gq complex). (**b**) Structure of G protein alpha subunit. It is consisted of Ras-like domain and α-helical domain (AHD). Please note that AHD is displaced in the GPCR-G protein complex structure in (**a**). (**c**) Approximate number of currently identified ligands, GPCRs and Gα families. (**d**) Diverse mechanism of GPCR-G protein activation. One GPCR can activate different G proteins, and one G protein can be activated by diverse GPCRs.

Much of the recent progress in GPCRs has been achieved after the crystal structure of β_2_-adrenergic receptor (β_2_AR) has been revealed^5, 6^. GPCRs share common structural features with seven-transmembrane (TM) helices (Fig. 1a). Upon ligand-binding, the seven TM helices undergo conformational changes, which promote the interaction of the receptor with downstream signaling molecules in cells^7^. The canonical downstream signaling molecules for GPCRs are heterotrimeric G proteins. G proteins consist of three subunits: Gα, Gβ, and Gγ (Fig. 1a). The Gα subunit consists of a Ras-like domain and α-helical domain’, and the nucleotide-binding pocket is located between these two domains (Fig. 1b). The Ras-like domain directly interacts with the receptor (Fig. 1b) and transduces signals to other effectors^8^. In the basal state, the Gα subunit is occupied by GDP and forms a heterotrimer with the Gβγ subunits. Active GPCRs act as guanine nucleotide exchange factors (GEFs) by releasing GDP from G proteins. Under physiological conditions, GTP is quickly inserted into the Gα subunit, which then dissociates from the receptor and Gβγ subunit.

G proteins are sub-categorized depending on the Gα subunit. In human, there are 16 Gα subunit genes, which are classified into four subtype families (G_s_, G_i/o_, G_q/11_, and G_12/13_) (Fig. 1c). Each subtype family of G proteins interacts with different downstream effector molecules to induce distinct cell responses^9^. In human, approximately 1000 ligands and 800 GPCRs have been identified (Fig. 1c). Therefore, one G protein subtype interacts with more than one receptor. Moreover, many receptors can couple to not only one G protein subtype but also more than one G protein subtypes (Fig. 1d).

In recent years there has been an increased research interest in explaining how GPCRs and G proteins recognize each other in a timely manner^10, 11^. However, complicated interaction patterns exacerbate to solve the selectivity problem (Fig. 1d). To determine the GPCR-G protein coupling selectivity, Flock et al*.* recently used an evolutionary approach^10^. They used the concepts of paralogue and orthologue conservation. If some positions in Gα protein have pronounced conservation of orthologue and paralogue, they are defined as the conserved group concerning their major function. Positions with a highly conserved orthologue and low paralogue conservation are defined as ‘selectivity barcode’. The authors identified the selectivity barcode positions critical for GPCR-G protein selectivity, which are available at GPCRdb (https://GPCRdb.org). This concept explaining selectivity determinants is innovative and persuasive. However, it is difficult to define the key components of selectivity determinants because (as discussed later in the paper) approximately 30–40% of amino acids are defined as selectivity barcodes (Supplementary Fig. S5a). Flock et al.^10^ mainly focused on GPCR-G protein interface regions, hence they could not fully explain the effects on selective binding of other residues outside the GPCR-G protein interface.

Here, we adopted another approach, sequence coevolution, to understand the GPCR-G protein coupling system. Sequence coevolution is used to predict protein contacts, folding network, allosteric network, functional sites, and other features^12^. It is similar to the evolutionary approach but differs in that it can coincidentally observe pairs of amino acids. Among the various coevolutionary approaches, statistical coupling analysis (SCA) defines functional clusters, also termed sectors. Sectors consist of highly coevolved residues^13^. We applied SCA to explain the selectivity and functionality of the GPCR-G protein coupling system.

## Results

**Figure 2:**
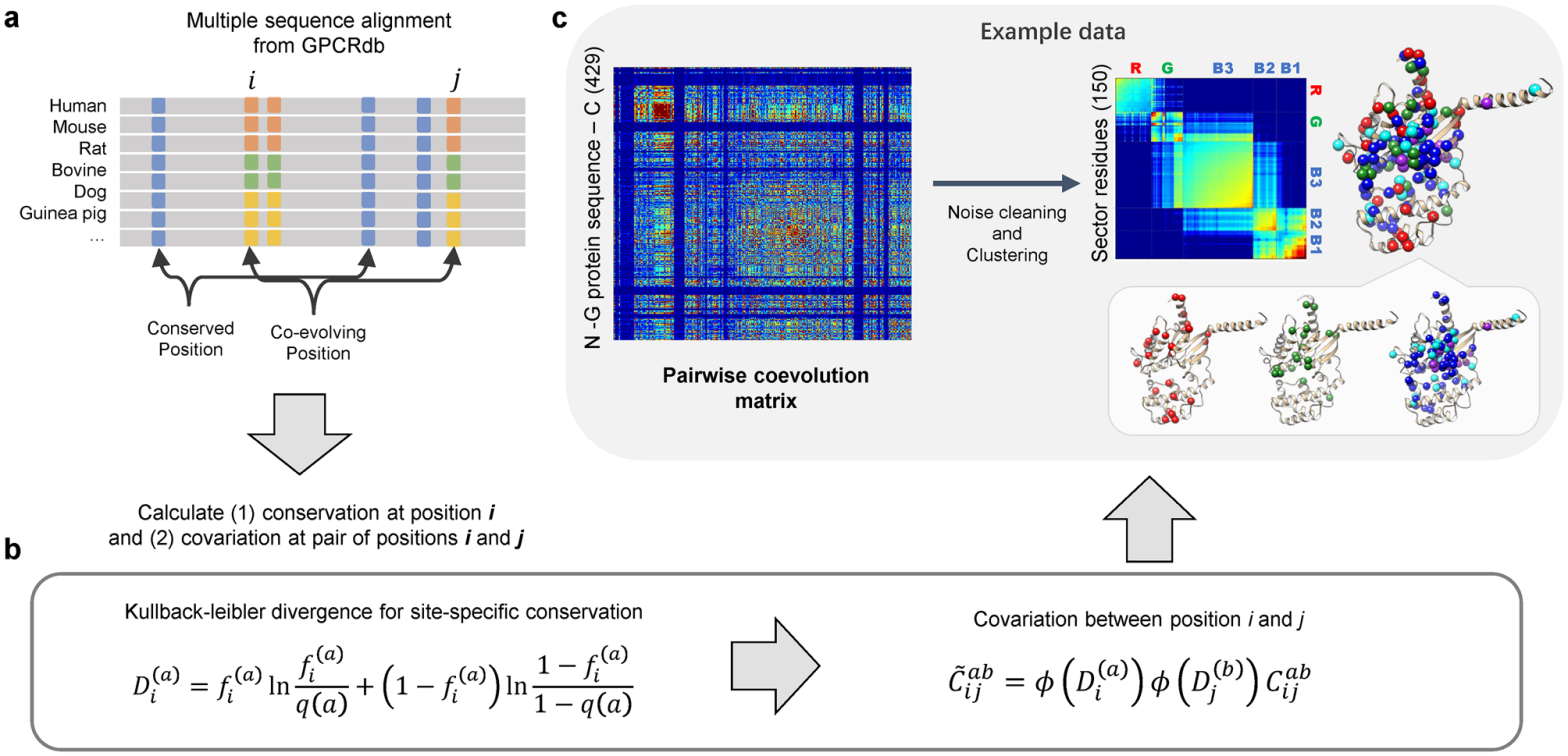
Overview of statistical coupling analysis (SCA). (**a**) Multiple sequence alignment is provided by GPCRdb (GPCRdb.org). (**b**) Calculation of Kullback–Leibler divergence for site-specific conservation and covariation between pairwise position. (**c**) Pairwise coevolution matrix is generated from (**b**). Then, through the noise cleaning and clustering process, sectors are defined.

To analyze the GPCR-G protein coupling system from a coevolutionary perspective, we applied the SCA which is one of the biophysical and bioinformatic tools. Originally, SCA was developed to measure the energetic coupling between residues in a protein^14^. It can now define functional coevolutionary units^13, 15^. This method measures conservation at position *i* and covariance between pairs of positions *i* and *j* in a protein multiple sequence alignment (Fig. 2a,b). After cleaning noise and clustering highly coevolving residues together from a pairwise coevolution matrix, a sector matrix is generated (Fig. 2c). Generally, a protein family with high sequence identity is a good target for SCA analysis. Therefore, Gα proteins are good candidates for SCA analysis. However, SCA has practical problems for analyzing GPCR families. GPCRs have numerous subfamilies, but they share very low sequence identity, even in the same subfamilies. The extremely low sequence identities make it difficult to interpret SCA results. We seek to choose GPCR subfamilies with relatively well-defined coupling G proteins and selected two subfamilies of Class A GPCRs as model receptors: 5-hydroxytryptamine (5-HT) receptors (HTRs) and dopamine receptors (DARs) (Supplementary Fig. S1).

### GPCRs have coevolved sectors and a common conserved sector

**Figure 3:**
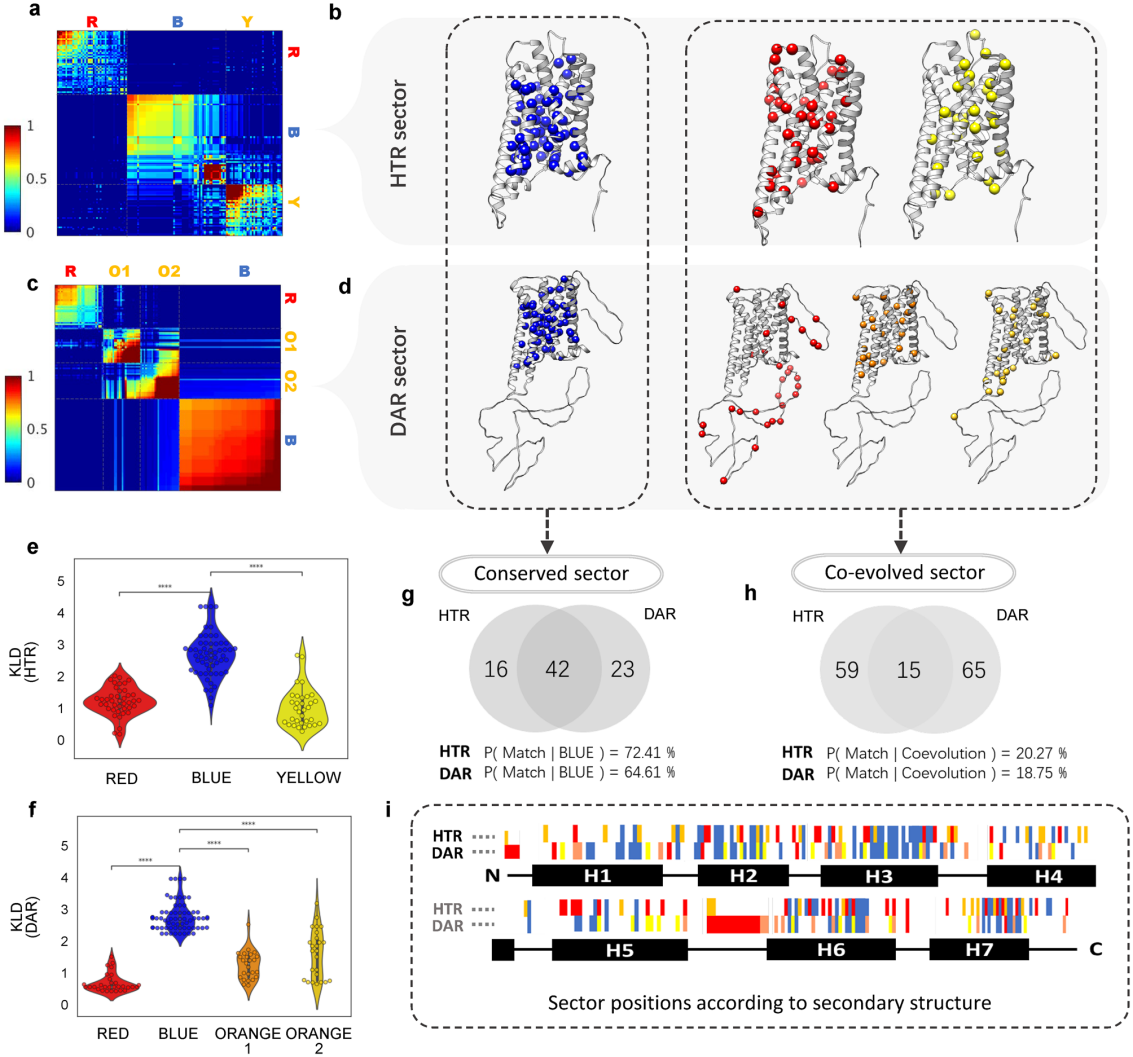
GPCRs have coevolved sectors and a common conserved sector. (**a**) Sector matrix of 5-HT receptor. (**b**) Structure representations of sectors of 5-HT receptor. (**c**) Sector matrix of DA receptor. (**d**) Structure representations of sectors of DA receptor. DAR-Orange1 is colored in orange, and DAR-Orange2 is colored in yellow. (**e**) Kullback–Leibler divergence of sectors of 5-HT receptor. Mann–Whitney test is performed between sectors. (****p ≤ 1.00e−04) (**f**) Kullback–Leibler divergence of sectors of DA receptor. Mann–Whitney test is performed between sectors. (****p ≤ 1.00e−04) (**g**) Venn-diagram of matched residue between HTR-Blue sector and DAR-Blue sector. (**h**) Venn-diagram of matched residue between coevolved sectors of HTR and DAR. (**i**) Sector positions of HTR and DAR according to secondary structure. At DAR row of (**i**), Orange color represents DAR-Orange1 and yellow represents DAR-Orange2.

We used multiple sequence alignments from GPCRdb because they provide well-labeled alignments using the Generic-GPCRdb numbering system by each receptor^16^. This labeling system makes comparisons much easier among the results of each receptor. We selected HTRs and DARs as model systems, as discussed above. Applying SCA and sector refinement algorithms to HTRs, we defined three sectors that were designated HTR-Red, HTR-Blue, and HTR-Yellow (Fig. 3a,b, Supplementary Table S1). Four DAR sectors were defined: DAR-Red, DAR-Blue, DAR-Orange1, and DAR-Orange2 (Fig. 3c,d, Supplementary Table S2). The total number of residues in the HTR and DAR sectors were 132 and 145, respectively.

To characterize the conservation of each sector, we examined the Kullback–Leibler divergence (KLD) of each sector (Fig. 3e,f) using the Mann–Whitney test. Higher KLD values meant more conservation. The HTR-Blue and DAR-Blue sectors consisted of more conserved residues than the other sectors. Generally, SCA sectors are coevolutionary groups^13^, but the HTR-Blue and DAR-Blue sectors are grouped by conservation. Therefore, we defined HTR-Blue and DAR-Blue sectors as conserved sectors. The others were defined as coevolution sectors.

HTR-Blue and DAR-Blue sectors were formed by highly conserved positions (Fig. 3e,f) and were found mainly in TM regions facing the inside of the receptors (Fig. 3b,d, Supplementary Fig. S2). These findings suggested that the blue sectors may be important to stabilize the TM structures. In addition, functionally important conserved motifs, such as DRY^17–23^, NPxxY(N,P,Y)^19–25^, CWxP(W,P)^19, 21, 23–25^, and PIF(P,I)^22, 23, 26^ were identified in the blue sectors (Supplementary Fig. S2). Moreover, conserved fingerprint residue^21^, disulfide bonds, and Na^+^ pockets were involved^21, 23, 27, 28^. Some ligand-binding pockets^23, 28^ and G protein coupling regions were also in the blue sectors^21^. These analyses indicated the involvement of the blue sectors in the conserved functions of GPCRs^17–20^.

The red sectors in HTR and DAR showed interesting correlations with the GPCR-G protein coupling patterns (Supplementary Fig. S1). The HTR-Red positions could be roughly divided into Gi-coupled receptors and Gq coupled receptors (Supplementary Fig. S1a and Supplementary Dataset S1). Similarly, residues in DAR-Red correlated with G protein selectivity which distinguish D1-like and D2-like subtypes (Supplementary Fig. S1b). The positions of HTR-Red were determined to be located in the intracellular regions of TM 3–7, intracellular loop 2 (ICL2), and helix 8 (Fig. 3b and Supplementary Fig. S2a). These regions have been suggested to be the major contact sites for G proteins^10^. The positions of the DAR-Red sector were mostly located in ICL3 (Fig. 3d and Supplementary Fig. S2b), which may selectively interact with downstream signaling proteins. Additionally, HTR-Red contained residues from the ligand-binding pocket and conserved motifs for GPCR activation (Fig. 3b and Supplementary Fig. S2a)^21^. These results support the suggestion that the red sectors are involved in G protein selectivity, which is discussed further below.

There were other sectors, such as HTR-Yellow, DAR-Orange1, and DAR-Orange2 sectors. HTR-Yellow belonged to the coevolutionary groups and it has lower KLD values than the blue sectors. (Fig. 3e). Interestingly, HTR-Yellow contained several ligand-binding residues (Fig. 3b, Supplementary Table S4, Supplementary Fig. S2a). The pattern of amino acid sequences in HTR-Yellow allowed further subdivision into HTR subtypes (Supplementary Fig. S1a, Supplementary Dataset S1). We analyzed mutation effects of HTR-yellow with mutant browser at GPCRdb (Supplementary Fig. S3). The results showed the residues of HTR-Yellow could make the changes to ligand binding potency and it was different for each position and subtypes. Therefore, we presumed that this sector may be related to the fine-tuning of ligand-binding responses and may regulate subtype specific reactions. The DAR-Orange1 and DAR-Orange2 sectors were also coevolved sectors (Fig. 3d), as they were less conserved than the blue sector. (Fig. 3f) The DAR-Orange1 and DAR-Orange2 positions clearly showed the subtype specific sequences (Supplementary Fig. S1b). Positions of DAR-Orange1 had D3-D4 specific amino acid pattern and positions of DAR-Orange2 had a D4-specific amino acid pattern (Supplementary Fig. S1b, Supplementary Dataset S1). Several positions from DAR-Orange1 and DAR-Orange2 were distributed at the ligand-binding residues in the intracellular regions of TM5 and TM6, ICL2, and conserved motifs that include the DRY, CWxP, and NPxxY motifs (Fig. 3d and Supplementary Table S4, Supplementary Fig. S2b). These analyses suggested that DAR-Orange1 and DAR-Orange2 might also be involved in GPCR-G protein coupling and subtype specific characteristics, although certain functions of DAR-Orange1 and DAR-Orange2 were ambiguous. These results affirmed that specific subtype characteristics could be checked using coevolutionary analysis.

We compared the sector positions of HTR and DAR using GPCRdb numbering systems. Comparing HTR-Blue to DAR-Blue, 70% of the positions matched (Fig. 3g,i). Almost of matched positions tended to be conserved in aminergic receptor family while some positions showed relatively low conservation at class A level (Supplementary Fig. S4). This results mat be caused by the differences between aminergic-wide and class A-wide conservation. This indicated that major functions have evolved through a group of residues that are conserved in each family of other GPCRs. However, coevolved sectors (red, yellow, oranges) had their own characteristics, and they shared only 20% of residues (Fig. 3h,i). The coevolved sectors showed interesting features at ligand–receptor interactions. We analyzed available crystal structures and their ligand interactions, then we found the specific receptor–ligand interactions (Supplementary Table S4, Supplementary Dataset 2). The common ligand-interactive residues were mostly blue sectors and ligand-specific interactive residues were mostly the coevolved sectors. This result may indicate the coevolved residues have modulated interactions to various ligands.

### Functional networks in G protein from recognition to signal transduction

**Figure 4:**
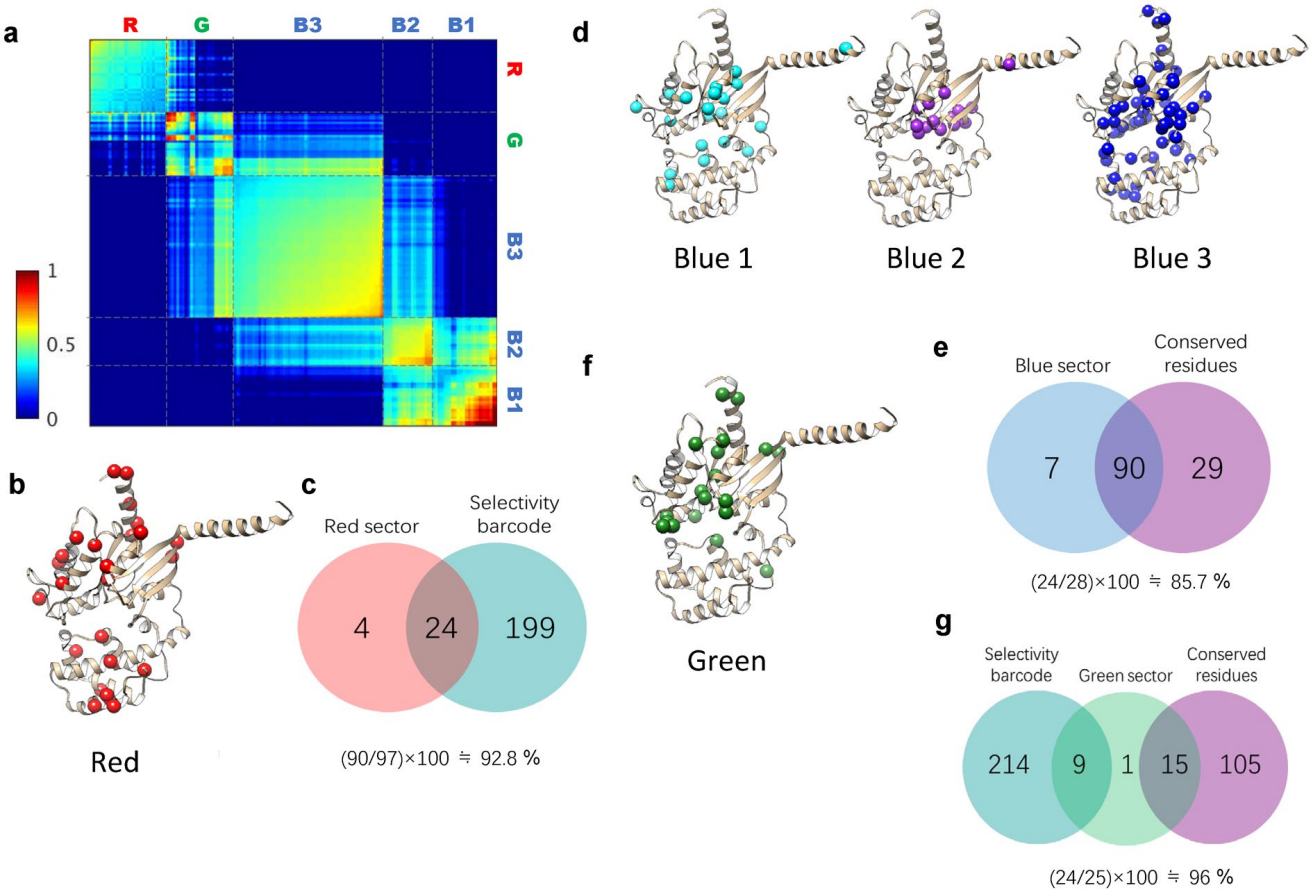
Functional networks in G protein from recognition to signal transduction. (**a**) Sector matrix of G protein. Three sectors (G-Red, G-Green, G-Blue) are existed and G-Blue sectors are subdivided into three subsectors (G-Blue1, G-Blue2, G-Blue3). (**b**) Structure representation of red sector. (**c**) Venn-diagram of matched residue between G-Red and selectivity barcode defined by previous work^10^. (**d**) Structure representation of blue sectors. (**e**) Venn-diagram of matched residue between G-Blue and conserved residues defined by previous work. (**f**) Structure representation of green sector. (**g**) Venn-diagram of matched residues among G-Green, Selectivity barcode and conserved residue. Close form of G protein structure is the modeling structure by Alhadeff et al.^34^.

The interaction between GPCRs and G proteins mostly occurs at the Gα subunit. Gα subunits, but not the Gβγ subunits, have been suggested to be the key protein for GPCR-G protein selectivity^29–31^. Therefore, we performed SCA with 16 Gα proteins. The Gα protein residues comprised three sectors (G-Red, G-Green, and G-Blue) (Fig. 4b and Supplementary Table S3). Total number of sector residues is 150. Most of these sectors were identified in the core of the Ras-like domain, with some in the α-helical domain and αN (Fig. 4 and Supplementary Fig. S5). The Ras-like domain has been suggested to have critical functions of Gα proteins, such as receptor binding, GTPase activity, and effector binding^29, 30, 32^. Thus, the identified sectors might be involved in G protein functions.

**Figure 5:**
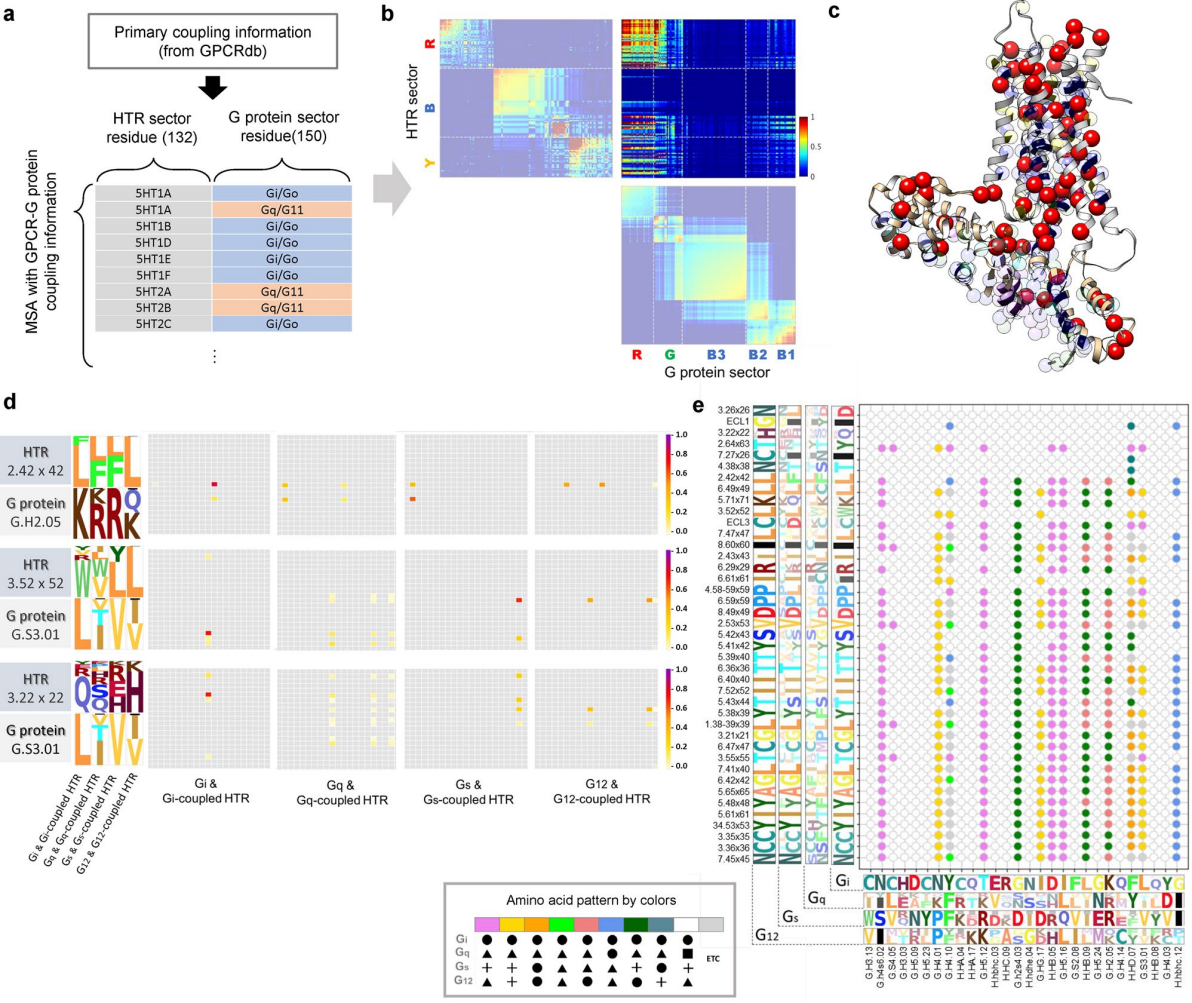
Red sectors are mediator between GPCR and G protein signaling. (**a**) Construction of new multiple sequence alignments for integrated analysis of GPCR-G protein. (**b**) Results of integrated SCA. Blurred matrices are original sector matrix of HTR and G protein. The cross section of each matrix is covariation between HTR sectors and G protein sectors. (**c**) Structure representation of HTR-Red and G-Red which have high coevolution signals. (**d**) Sequence logo and amino acids patterns of HTR-Red and G-Red interaction. In amino acid pattern figure, x, y axis represent the amino acid order of three letter code alphabetically from top to bottom, left to right. (one letter code order: ARNDCQEGHILKMFPSTWYV-, -represents gap). (**e**) Amino acid pattern analysis between HTR-Red and G-Red residues. Patterns of each color are explained in legend. Same shape in each column represents similar distribution of amino acids’ pair.

The majority of G-Red sector residues were positioned at the interface with GPCRs (e.g. β1, β2 loop, C-terminal part of α3 and α4, and α5), although some were positioned in the α-helical domain (Fig. 4b and Supplementary Fig. S5b). Flock et al. defined the ‘selectivity barcode’ that determines GPCR-G protein selectivity using evolutionary concepts (Supplementary Fig. S5a), and identified 25 CGN positions in these barcodes that contacted the receptor^10^. Interestingly, most of the G-Red sector positions (24 of 28 positions) overlapped with the selectivity barcode suggested by Flock et al. (Fig. 4c and Supplementary Fig. S5)^10^. This results suggested that G-Red might serve as a determinant of GPCR-G protein selectivity. This suggestion is supported by the analysis described in Fig. 5. Flock et al.^10^ defined selectivity determinant residues using evolutionary concepts resulting in approximately 30–40% of amino acids defined as selectivity barcodes (Supplementary Fig. S3a). The present results were a marked improvement in selecting more specific positions for selectivity determinants.

Concerning the functions of sectors, when compared with previous findings^10^, the G-Blue sector was the most conserved sector in the Gα proteins (Fig. 4d,e). More specifically, the G-Blue sector comprised three subsectors: G-Blue1, G-Blue2, and G-Blue3. These subsectors contained many key residues for essential functions (Fig. 4a,d, and Supplementary Fig. S3b). Positions of G-Blue1 contain switch 1, 2, and 3 regions that regulate G protein activation status by changing their conformation depending on the binding GDP or GTP (Fig. 4d and Supplementary Fig. S5b)^31^. Residues of the G-Blue2 sector were mostly located around α1 helix (Fig. 4d and Supplementary Fig. S5b), which is related to stabilization of GDP binding^33^. Residues from G-Blue3 also contained residues from the switch 1, 2, and 3 regions (Fig. 4d and Supplementary Fig. S5b). Moreover, the majority of G-Blue3 residues were located in the regions that stabilized the basal conformation of the Gα proteins, which include hydrophobic residues at G.H5.07 and G.H.5.08 and residues within the β-sheets, inter-domain interface, and TCAT motif (Fig. 4d and Supplementary Fig. S5b). Interestingly, G-Blue3 was also present at the C-terminus of α5 helix (Fig. 4d and Supplementary Fig. S5b), where the Gα protein formed a major interface with a receptor (Fig. 1a)^8^ and G-Blue3 bridged to the G-Red sector in the sector matrix (Fig. 4a). Based on these data, it can be suggested that, among G-Blue sectors, G-Blue3 is the first to receive the recognition signal from G-Red.

Lastly, the G-Green sector seems to be an intermediate group between G-Red and G-Blue. The sector matrix revealed that G-Green sector was located between the G-Red and G-Blue sectors (Fig. 4a). The G-Green sector residues had G-Red-like and G-Blue-like properties (Fig. 4f, Supplementary Fig. S5c). More specifically, the residues that overlapped with the selectivity barcode or conserved residues were plotted on the snake map (Fig. 4g and Supplementary Fig. S5c). The conserved residues found in G-Green (Supplementary Fig. S5c, purple) were mostly involved in stabilizing the conformation of the Gα protein. The selectivity barcode residues within G-Green (Supplementary Fig. S5c, green) were divided into two positions. Five of eight were located in the upper part of the Gα protein, making them potential receptor binding residues. The remaining three were located near the nucleotide-binding pocket. Therefore, we presumed that the signals from GPCRs were somehow received in the G-Red sector and transduced to G-Blue to perform the actual functions of G protein, which is mediated by G-Green sectors.

### Red sectors mediate between GPCR and G protein signaling

To integrate the analyses of GPCRs and Gα proteins, we built a new multiple sequence alignment with coupling information. First, we used the sector positions of GPCRs and Gα proteins to study the relationship between GPCR coevolution and Gα protein coevolution. We concatenated the HTR sector to the Gα protein sector with known selectivity information in GPCRdb (Fig. 5a). For example, the HTR1A family interacted with the Gi and Gq family. Therefore, we repeated the HTR1A sequence twice and integrated one with Gαi and the other with Gαq. HTR1B coupled with only Gi. Therefore, we simply integrated HTR1B residues with Gαi. After repeating this process for all subtypes of HTR, we ran the SCA process and obtained a positional coevolution matrix.

Integrated SCA revealed a strong correlation signal between the HTR-Red and G-Red sectors (Fig. 5b,c). This result is consistent with a previous interpretation of the sectors, because each is suggested to be involved in selective recognition between GPCRs and G proteins (Figs. 3, 4). In other words, the HTR-Red and G-Red sectors were strongly coevolved and played a role as selectivity determinants. Additionally, G-Red also weakly coevolved with some positions in HTR-Blue or HTR-Yellow, and these make minor correlation signals (Fig. 5b).

To better understand the coevolution and selectivity between the red sector of GPCRs and the red sector of Gα proteins, we analyzed the pattern of coevolving amino acids. The results of HTR-Gα protein pattern analysis revealed the pairwise pattern of Gα protein-specific amino acids (Fig. 5d). For example, the pairwise frequency of amino acids between residue 2.42 in HTR and G.H2.05 in Gα protein are shown in the first row of Fig. 5d. Each 21 × 21 matrix in Fig. 5d represents Gαi, Gαq, Gαs, and Gα12 coupled sequence patterns. Gαi-coupling and Gα12-coupling had similar amino acid patterns. Likewise, Gαq-coupling and Gαs-coupling showed similar amino acid patterns. These results are visually represented as sequence logos in Fig. 5d. Amino acid frequencies between HTR 3.52 and G protein G.S3.01 are shown in the middle of the rows (Fig. 5d). While Gαs coupling and Gα12 coupling had similar amino acid patterns, the patterns of Gαi-coupling and Gαq-coupling were different and distinct from the others. The last row shows that the patterns of GPCR-Gα protein coupling are all different types between HTR 3.22 and Gα protein G.S3.01 (Fig. 5d). We examined all possible pairs between GPCR and G protein sectors and represented patterns with color coding (Fig. 5e). Distinguishing patterns of amino acid pairs were evident between HTR-Red and G-Red (Fig. 5e). Similar results were also found at DAR (Supplementary Fig. S6a,b). DAR-Red and G-Red were strongly coevolved (Supplementary Fig. S6a). Moreover, the other coevolved sectors, DAR oranges, were also correlated to DAR-Red. Most of DAR residues had different amino acid compositions depending on their G protein selectivity (Supplementary Fig. S6b). Amino acid patterns of DAR-G protein were simpler than HTR cases, because DAR does not primarily couple to Gα12. According to the results, we could conclude that intra-coevolution units make inter-coevolution with other proteins in case of GPCR-G protein. These are the most relevant evidence for coevolutionary sectors as a selectivity determinant.

When we analyzed the GPCR-Blue and G-Blue interactions in both receptors, most of the residue pairs had unified amino acids in four different Gα subtypes (Supplementary Fig. S7a,b). These results affirm that the blue sectors are conserved through subtypes of GPCRs and Gα. Compared to the analysis of red sectors, its interactions were less related to GPCR-G protein selectivity. Furthermore, the results of pattern analysis showed differences between G-Blue1 and G-Blue3, which were not clearly distinct from the SCA results (Supplementary Fig. S7a). Residues of G-Blue1 distinguished amino acids in G12 subtypes and different selectivity patterns for GPCR coupling.

## Discussion

GPCR-G protein coupling selectivity has been an active research topic. Extensive studies on three-dimensional GPCR-G protein complex structures analyzed by X-ray crystallography or cryo-electron microscopy (cryo-EM)^32, 35^ have failed to deduce the precise coupling selectivity mechanism. This is probably because most of these structures are GDP-released final state of GPCR-G protein complex structures. Recently, it was suggested that step-wise conformational changes occur during GPCR-G protein coupling, and that the early-stage structure is different from the structures observed from the currently available X-ray or cryo-EM structures^36, 37^. Therefore, there have been attempt to solve this selectivity question with evolutionary approach, experimental approach, and various computational simulations^10, 11, 38^. However, it remains still elusive to explain. Therefore, we tried to explain this issue and fill the missing parts of the selectivity mechanism using a coevolutionary approach.

SCA is a popular tool for coevolutionary analysis, but it is difficult to apply to proteins with low sequence identity, such as GPCR because multiple sequence alignments (MSA) with low sequence identity makes it difficult to separate coevolving positions from evolutionary random sequences. To overcome this problem, we selected a unit of the receptor sub-family. This approach enabled the analysis of model GPCRs and it could be possible to extend further research about general GPCR family. In addition, we built the input alignments with coupling information to consider GPCR-Gα protein binding selectivity (Fig. 5a and Supplementary Fig. S6a). By reflecting the selectivity of MSA, residue pairs for selective GPCR-Gα coupling were shown through SCA analysis.

The most progress compared to the study of Flock et al.^10^ is that the current study provided selectivity determinants within the receptors (HTR-Red and DAR-Red) and narrowed the selectivity determinants within the Gα proteins (28 residues of G-Red sector). Red sector residues from GPCRs were subdivided according to the coupling G proteins (Supplementary Fig. S1). Strong coevolving signals were evident between the red sectors of GPCRs and Gα proteins (Fig. 5b and Supplementary Fig. S6a). Furthermore, Gα protein-specific amino acid patterns coevolved together (Fig. 5e and Supplementary Fig. S6b). Among them, some residues were not located in binding interface, but these residues may be related with allostery effects. Similar results were obtained for HTR and DAR. Thus, although we did not analyze the class A GPCR family, defining the selectivity pattern related to coevolution may be generalized to class A GPCRs.

G-Red residues are located in both Ras-like and α-helical domains (Supplementary Fig. S5b). Within Ras-like domain, G-Red residues are mainly localized at reported receptor-contact sites such as β2/β3, α2/β4, α4/β6 loops and α5^33, 39–45^ and potential contact sites, such as α3, αG, and α4. These sites may contact a receptor when the receptor has a long ICL3, although long ICL3 is often not resolved or is truncated in all reported GPCR-G protein structures. Other residues within the Ras-like domain are α2 (one residue) and β4 (one residue). The potential involvement of these residues in GPCR-G protein coupling selectivity should be further investigated.

The identification of G-Red within α helical domain was unexpected because it does not directly interact with receptors. The role of the α-helical domain has not been well studied. The results from several studies indicated that the domain may influence GTP hydrolysis activities depending on Gα subtypes^30, 46, 47^ and might regulate effector^30, 48^. Generally, residues in α-helical domains have more divergent sequences than the Ras-like domain. Therefore, the suggested ten G-Red residues within the α-helical domain may be involved in GPCR-G protein selectivity and also in coevolution with other downstream effectors. These possibilities require further investigation.

In addition to the GPCR-G protein coupling selectivity determinants, SCA revealed other functional sectors. The blue sectors of each target protein (HTR-Blue, DAR-Blue, G-Blue) were highly conserved compared to other sectors (Figs. 3e,f and 4e). These sectors are critical role in their conserved functions (e.g., ligand-binding for GPCRs and GTP-mediated G protein activation for Gα proteins) and conformational integrity (Supplementary Figs. S2, S5). The results of the sector analysis also implied that G-Green may be an intermediary between the G-Red and G-Blue sectors (Fig. 4a) and that HTR-Yellow and DAR-Orange sectors are assumed to regulate receptor subtype specific characteristics because of the features of the sequence alignments (Supplementary Fig. S1).

In conclusion, the significance of our study is the suggested clarification of the GPCR-Gα selectivity mechanism and functional sectors using coevolutionary analysis. The actual roles of these sectors need to be confirmed with further experimental approaches. The coevolutionary analysis approach adopted in this study could be extended to class A GPCRs and for other molecules, such as arrestin, G protein-coupled receptor kinases (GRKs), or regulators of G protein signaling (RGSs). Moreover, the finding that subtype specific sequences can be extracted by SCA highlights the possibility of application in the pharmacological field for receptor subtype-selective targeting within the same subfamilies. The current research on coevolving sequences shows promise for systematic studies on GPCR-driven signaling.

## Materials and methods

### Statistical coupling analysis (SCA)

The SCA 5.0 toolbox used for SCA was implemented in MATLAB (The MathWorks, Inc., Natick, MA, USA). SCA was originally developed to measure evolutionary covariance between protein positions^13–15^. All MSA data were obtained from GPCRdb (GPCRdb.org). We selected sequences from all species provided in the database. In the major processing step, the positional conservation level was obtained using the KLD at position *i* and covariance between positions *i* and *j*. After these calculations, we removed statistical and historical noise and defined clusters (sectors) that consisted of highly coevolved positions. The details of the measurement procedure are described in a previous study^13^. Details of methods are in Supplementary Appendix.

### Sector refinements

SCA could exclude high coevolved positions or involve relatively weak positions while defining sectors with eigenvectors. Therefore, we added sector refinement steps to permit a similarity comparison. Some residues in non-sector positions with pronounced similarity with the sector were newly defined as a sector. In addition, some residues in the sector that displayed low similarity were discarded. The detailed process is described in Supplementary Fig. S8.

### GPCR-G protein integrated SCA

We constructed an integrated multiple sequence alignment to measure the coevolution between GPCR and G protein with previously defined sector positions. To reflect actual biological relationships, we considered the GPCR-G protein coupling pattern during MSA construction. For instance, HTR1A primarily interacts with Gi/Go and Gq/G11, generating HTR1A-Gi/o and HTR1A-Gq/o. We repeated this for every subtype of the target receptor family. The reference for coupling patterns was taken from GPCRdb, especially the merged one from two different data sources (Guide To Pharmacology DB, Inoue et al*.*^11^) We only considered primary coupling.

SCA processes were run with integrated alignments. However, we did not consider the noise cleaning step and did not drop any positions for clustering because we wanted to observe covariances between the already defined sector. Validation process for coevolution between GPCR and G proteins is in Supplementary information.

### GPCR-G protein pattern analysis

The HTR-Red and G-Red interaction represented in Fig. 5d,e is considered as an example. First, we constructed four MSA according to the G protein coupling pattern (for example, Gi-coupled HTR sequences combined with Gi sequences). The alignments only involved sector positions of HTR (HTR-Red, 41 positions) and G proteins (G-Red, 28 positions).

For each pair of HTR-Red and G-Red (41 × 28), four sets of pairwise amino acid frequencies were counted and 21 × 21 matrices (20 amino acid plus gaps) were generated. Plots in Fig. 5d are examples of 21 × 21 matrices. Then, we converted the 21 × 21 matrices to 1 × 441 vectors to generate, four sets of 1 × 441 vectors representing each of the HTR-G protein subtype coupling. The inner products between the four sets of vector groups were calculated to measure their similarities. There were four G protein subtypes and six possible combinations of inner products between them. We finally obtained six inner product values for each pair of HTR and G proteins (for example, group 1 consisted of [HTR1-Gi1]∙[HTR1∙Gq1], [HTR1-Gi1]∙[HTR1∙Gs1], [HTR1-Gi1]∙[HTR1∙G121], [HTR1-Gq1]∙[HTR1∙Gs1], [HTR1-Gq1]∙[HTR1∙G121], [HTR1-Gs1]∙[HTR1∙G121]). The threshold value to assess whether the patterns were similar or not was 0.6, and classified into 15 patterns. For instance, if all inner products values are under the threshold, four subtypes of HTR-G protein are having different patterns. We repeated this process to all pairs of HTR-Red and G-Red (41 × 28). (Supplementary Fig. S9).

### Structure and graphic representation

The structures used for the GPCR-G protein combined form and GPCR structure were downloaded from the GPCRdb modeled structure list. We selected a 5HT2B-Gq coupled structure for HTR. For DAR, we used the drd2 modeled structure from GPCRdb. However, the drd2 structure from GPCRdb lost many parts of their ICL3. Therefore, we constructed the ICL3 of the DAR using MODELLER^49^. The G protein structure shown in Figs. 1 and 3 was provided is by Alhadeff et al*.* (active-closed-in form)^34^. To map sectors to structure, we first needed to align the sequence used for SCA and the sequence of the structure. MUSCLE (https://www.ebi.ac.uk/Tools/msa/muscle) alignment web server^50^ was used for the alignment. All protein structure representations were visualized using UCSF chimera 1.14 software^51^.

### Visualization and statistics

Visualization of KLD (Fig. 3e,f) and the data in Fig. 5d,e were visualized using the Python package (matplotlib^52^ and seaborn^53^). Statistical validations were performed using the Python package (statannot). The sequence logo plots in Fig. 5 were drawn using the Logomaker package. The remaining plots and matrices were generated using the SCA MATLAB code.

## Supplementary Information

Supplementary Dataset 1.

Supplementary Dataset 2.

Supplementary Information.
