# Supplementary material for: Coevolution underlies GPCR-G protein selectivity and functionality
Source: Sci Rep. 2021 Apr 12;11:7858. doi: 10.1038/s41598-021-87251-6 (PMC8041822; doi:10.1038/s41598-021-87251-6)
Supplement: Supplementary file 3 — Supplementary Information. [file 41598_2021_87251_MOESM3_ESM.pdf]

Supplementary Information for
Coevolution underlies GPCR-G protein selectivity and functionality

Min Jae Seo, Joongyu Heo, Kyunghui Kim, Ka Young Chung & Wookyung Yu*

Wookyung Yu
Email: wkyu@dgist.ac.kr

This PDF file includes:

Supplementary tables S1 to S4
Supplementary figures S1 to S9
Supplementary text – Details of methods
References

Other supplementary materials for this manuscript include the following:

Supplementary Dataset S1
Supplementary Dataset S2

Supplementary Table S1. HTR sector positions

HTR Sector	*Positions (GPCRdb numbering)*
RED	1.38-39x39, 2.42x42, 2.43x43, 2.53x53, 2.64x63, ECL1(MSA pos 293), 3.21x21,3.22x22, 3.26x26, 3.35x35, 3.36x36, 3.52x52, 3.55x55, 34.53x53, 4.38x38, 4.58-59x59, 5.38x39, 5.39x40, 5.41x42, 5.42x43, 5.43x44, 5.48x48, 5.61x61, 5.65x65, 5.71x71, 6.29x29, 6.36x36, 6.40x40, 6.42x42, 6.47x47, 6.49x49, 6.59x59, 6.61x61, ECL3(MSA pos 802), 7.27x26, 7.41x40, 7.45x45, 7.47x47, 7.52x52, 8.49x49, 8.60x60,
BLUE	1.50x50, 1.53x53, 12.50x50, 2.40x40, 2.41x41, 2.45x45, 2.46x46, 2.47x47, 2.50x50, 2.56x551, 2.57x56, 2.59x58, 23.50x50, 3.25x25, 3.32x32, 3.37x37, 3.39x39, 3.40x40, 3.42x42, 3.43x43, 3.44x44, 3.46x46, 3.48x48, 3.49x49, 3.50x50, 3.51x51, 3.53x53, 3.54x54, 4.42x42, 4.46x46, 4.50x50, 4.57x57, 4.59-60x60,<br><br>45.50x50, 5.47x47, 5.50x50, 5.58x58, 6.30x30, 6.32x32, 6.35x35, 6.37x37, 6.38x38, 6.44x44, 6.48x48, 6.50x50, 6.51x51, 6.52x52, 6.53x53, 7.40x39, 7.42x41, 7.43x42, 7.46x46, 7.49x49, 7.50x50, 7.53x53, 8.47x47, 8.53x53, 8.54x54
YELLOW	N-term(MSA pos 209), 1.32-33x32, 1.46x46, 1.54x54, 12.49x49, 12.51x51, 2.52x52, 2.58x57, 2.61x60, 23.49x49, 3.28x28, 3.38x38, 34.52x52, 34.54x54, 4.49x49, 4.53x53, 4.54x54, 4.61-62x62, 4.62-63x63, ECL2(MSA pos 381), 5.54x54, ICL3(MSA pos 453, 460, 467, 749), 6.33x33, 6.46x46, 7.33x32, 7.36x35, 7.38x37, 7.44x43, 7.48x48, 7.54x54

Supplementary Table S2. DAR sector positions

DAR Sector	*Positions (GPCRdb numbering)*
RED	N-term(MSA pos 4, 8, 11, 12, 21), 1.34x34, 12.48x48, 2.41x41, ECL1(MSA pos 108), 4.41x41, ICL3(MSA pos287, 288, 290, 294, 297, 301, 303, 304, 308, 310, 312, 321, 325, 327, 364, 374, 406, 408), ECL3(MSA pos 466), 7.48x48
ORANGE1	1.39x39, 1.40x40, 1.59x59, 3.24x24, 3.27x27, 3.28x28, 3.52x52, 34.51x51, 34.57x57, 4.53x53, 4.56x56, 5.71x71, ICL3(MSA pos 271, 410, 421), 6.24x24, 6.26x26, 6.28x28, 6.37x37, 6.43x43, 6.53x53, 6.61x61, 7.38x37, 7.51x51, 8.59x59
ORANGE2	1.36x36, 1.56x56, 2.48x48, 2.53x53, 2.58x57, 2.60x59, 23.52x52, 3.29x29, 3.35x35, 3.41x41, 3.51x51, 4.38x38, 4.57x57, 5.48x48, 5.57x57, 5.61x61, 5.62x62, 5.64x64, 5.69x69, 6.31x31, 6.45x45, 6.49x49, 6.60x60, 7.35x34, 7.55x55
BLUE	1.42x42, 1.43x43, 1.49x49, 1.50x50, 1.52x52, 1.53x53, 1.54x54, 12.50x50, 2.39x39, 2.40x40, 2.45x45, 2.46x46, 2.47x47, 2.50x50, 2.51x51, 2.54x54, 2.56x551, 2.57x56, 2.59x58, 2.65x64, 23.50x50, 3.25x25, 3.32x32, 3.34x34, 3.37x37, 3.38x38, 3.39x39, 3.40x40, 3.42x42, 3.43x43, 3.44x44, 3.46x46, 3.47x47, 3.49x49, 3.50x50, 3.53x53, 34.50x50, 34.53x53, 4.50x50, 4.52x52, 4.60-61x61, 45.50x50, 5.42x43, 5.43x44, 5.46x461, 5.47x47, 5.50x50, 5.58x58, 6.30x30, 6.32x32, 6.42x42, 6.44x44, 6.47x47, 6.48x48, 6.50x50, 6.51x51, 6.52x52, 7.40x39, 7.42x41, 7.45x45, 7.46x46, 7.49x49, 7.50x50, 7.52x52, 7.53x53

Supplementary Table S3. G protein sector positions

G Protein Sector	*Positions (GPCRdb numbering)*
RED	H.HA.04, H.HA.17, H.HB.05, H.HB.08, H.HB.09, H.hbhc.03, H.hbhc.12, H.HC.09, H.HD.07, H.hdhe.04, G.S2.08, G.S3.01, G.H2.05, G.h2s4.03, G.S4.05, G.H3.03, G.H3.13, G.HG.17, G.H4.01, G.H4.03, G.H4.10, G.H4.14, G.h4s6.02,G.H5.09, G.H5.12, G.H5.16, G.H5.23, G.H5.24
GREEN	G.S1.02, G.s1h1.02, H.hdhe.02, H.HE.01, H.hehf.04, G.s2s3.01, G.S3.03, G.S3.05, G.H2.01, G.H2.02, G.h2s4.05, G.S4.02, G.S4.04, G.S4.06, G.s4h3.05, G.s4h3.07, G.s4h3.08, G.s4h3.11, G.H3.14, G.H3.18, G.S5.02, G.HG.03, G.H4.15, G.H5.19, G.H5.21,
BLUE1	G.HN.09, G.HN.34, G.S1.04, G.S1.06, G.h1ha.03, H.HA.02, H.HA.26, H.HD.06, H.hdhe.05, H.HE.02, H.hehf.07, H.HF.06, G.H2.03, G.H2.06, G.H2.08, G.h2s4.04, G.S4.03, G.s4h3.04, G.H3.08, G.hgh4.03, G.H4.06, G.H4.11, G.s6h5.02, G.s6h5.05
BLUE2	G.HN.01, G.HN.47, G.S1.05, G.S1.07, G.s1h1.01, G.s1h1.03, G.s1h1.04, G.s1h1.05, G.s1h1.06, G.H1.01, G.H1.02, G.H1.03, G.H1.06, G.H1.07, G.H1.08, G.H1.10, G.H1.12, H.HA.14
BLUE3	H.HA.25, H.HC.10, H.HC.11, H.hchd.01, H.HD.02, H.HD.09, H.HD.12, H.hdhe.03, H.HE.04, H.HE.06, H.hehf.06, H.HF.03, H.HF.05, G.hfs2.02, G.hfs2.05, G.hfs2.07, G.S2.01, G.S2.03, G.S2.06, G.S3.07, G.S3.08, G.s3h2.01, G.s3h2.02, G.s3h2.03, G.H2.04, G.H2.07, G.H2.10, G.h2s4.01, G.s4h3.02, G.s4h3.10, G.s4h3.15, G.H3.01, G.H3.04, G.H3.05, G.H3.09, G.H3.12, G.S5.01, G.S5.04, G.S5.05, G.S5.06, G.S5.07, G.s5hg.01, G.HG.02, G.HG.06, G.HG.07, G.S6.04, G.s6h5.01, G.s6h5.03, G.H5.01, G.H5.03, G.H5.07, G.H5.08, G.H5.13, G.H5.20, G.H5.25

Supplementary Table S4. Ligand-receptor interactions of sector residues

Function	Ligand (PDB code)	Positions	
		Specific	Common
**5-HT$_{1B}$**			
Agonist	Dihydroergotamine (4IAQ)	2.64x63, 7.36x35	3.36x36, 5.42x43, 3.32x32, 3.37x37, 3.40x40, 6.48x48, 6.51x51, 6.52x52, 7.43x42, 3.28x28
	Donitriptan (6G79)	5.39x40, 5.43x44	
Selective Agonist	Ergotamine (4IAR, 7C61)	2.64x63, 5.38x39	
Inverse Agonist (6WH4 fig. 3c)	CHEMBL428892 (5V54)	5.39x40, 5.43x44, 5.47x47	
**5-HT$_{2A}$**			
Agonist	25-CN-NBOH (6WHA)	2.53x53, 5.39x40, 5.42x43, 3.28x28	3.36x36, 3.32x32, 3.37x37, 6.48x48, 6.51x51, 6.52x52, 7.43x42
Arrestin Selective Agonist	Lysergide (6WGT)	2.61x60, 5.38x39, 5.39x40, 5.42x43, 5.43x44	
Antagonist	Risperidone (6A93)	2.61x60, 3.40x40, 6.44x44, 3.28x28	
	Zotepine (6A94)	5.39x40, 5.42x43, 5.43x44, 3.40x40, 6.44x44, 3.28x28	
Inverse Agonist	CHEMBL428892 (6WH4)	5.39x40, 5.42x43, 5.43x44, 3.40x40, 6.44x44, 3.28x28	
**5-HT$_{2B}$**			
Agonist	Methylergonovine (6DRY)	3.37x37, 7.36x35	3.36x36, 5.39x40, 3.32x32, 6.48x48, 6.51x51, 6.52x52, 7.43x42
Arrestin Selective Agonist	Ergotamine (4IB4, 4NC3, 5TUD)	6.59x59, 3.37x37, 3.40x40, 3.28x28, 7.36x35	
	Lysergide (5TVN)	3.37x37, 3.28x28, 7.36x35	
Gq Selective Agonist	LY266097 (6DS0)	2.64x63, 7.40x39, 2.61x60, 3.28x28, 7.36x35	
Antagonist	Lisuride (6DRX)	3.37x37, 3.28x28	
	Methysergide (6DRZ)	3.37x37, 7.36x35	
**5-HT$_{2C}$**			
Selective Inverse Agonist	Ritanserin	3.36x36, 5.39x40, 5.42x43, 5.43x44, 3.32x32, 3.36x36, 3.37x37, 3.40x40, 5.47x47, 6.48x48, 6.51x51, 6.52x52, 7.43x42, 2.61x60, 3.28x28, 7.36x35	
**D$_3$**			
Selective Antagonist	Eticlopride (3PBL)	3.32x32, 3.37x37, 5.42x43, 5.43x44, 5.46x461, 6.48x48, 6.51x51, 6.52x52, 2.61x60, 3.28x28, 3.29x29, 7.35x34	
**D$_4$**			
Antagonist	Nemonapride (5WIU, 5WIV)	2.57x56, 23.50x50, 3.32x32, 3.37x37, 45.50x50, 5.42x43, 5.43x44, 6.48x48, 6.51x51, 6.52x52, 3.28x28, 2.60x59, 3.29x29	

Words color: HTR: Red, Blue, Yellow Sector Residue; DAR: Blue, Orange Sector Residue

a. HTR

Sector		1A	1B	1D	1E	1F	2A	2B	2C	4R	5A	6R	7R
G protein coupling	Gi	Primary	Primary	Primary	Primary	Primary	Secondary	Secondary	Primary	Primary	Primary	Secondary	Secondary
	Gq	Primary	Secondary	Secondary	-	-	Primary	Primary	Primary	Secondary	Secondary	Secondary	Primary
	Gs	-	-	-	-	-	-	-	-	Primary	-	Primary	Primary
	G12	-	-	-	-	-	-	-	Secondary	Secondary	-	Secondary	Primary
Amino acids pattern	Blue	A	A	A	A	A	A	A	A	A	A	A	A
	Red	A1	A1	A1	A1	A1	B	B	B	C	A2	A3	A4
	Yellow	A	B1	B2	B3	B3	B4	B4	B4	C	D	E	F

b. DAR

Sector		D1	D2	D3	D4	D5
G protein coupling	Gi	Secondary	Primary	Primary	Primary	Secondary
	Gq	Primary	-	-	-	Secondary
	Gs	Primary	-	-	-	Primary
	G12	-	-	-	-	Secondary
Amino acids pattern	Blue	A	A	A	A	A
	Red	A	B	C	D	A
	Orange1	A1	A2	B	C	A1
	Orange2	A1	A2	A2	B	A1

Supplementary figure S1. Subtype classification of HTR and DAR using coevolved sectors. GPCR-G protein coupling information from GPCRdb. (Using merged source: Data from Guide to Pharmacology and *Inoue et al.*[1] Amino acid pattern depending on receptor subtypes are marked as alphabetical symbol intuitively. In each sector rows, same alphabetical symbol represents same amino acids patterns. Same alphabetical symbol and different numbers represents similar but not same amino acid patterns. Entire amino acids patterns are in Dataset S1.

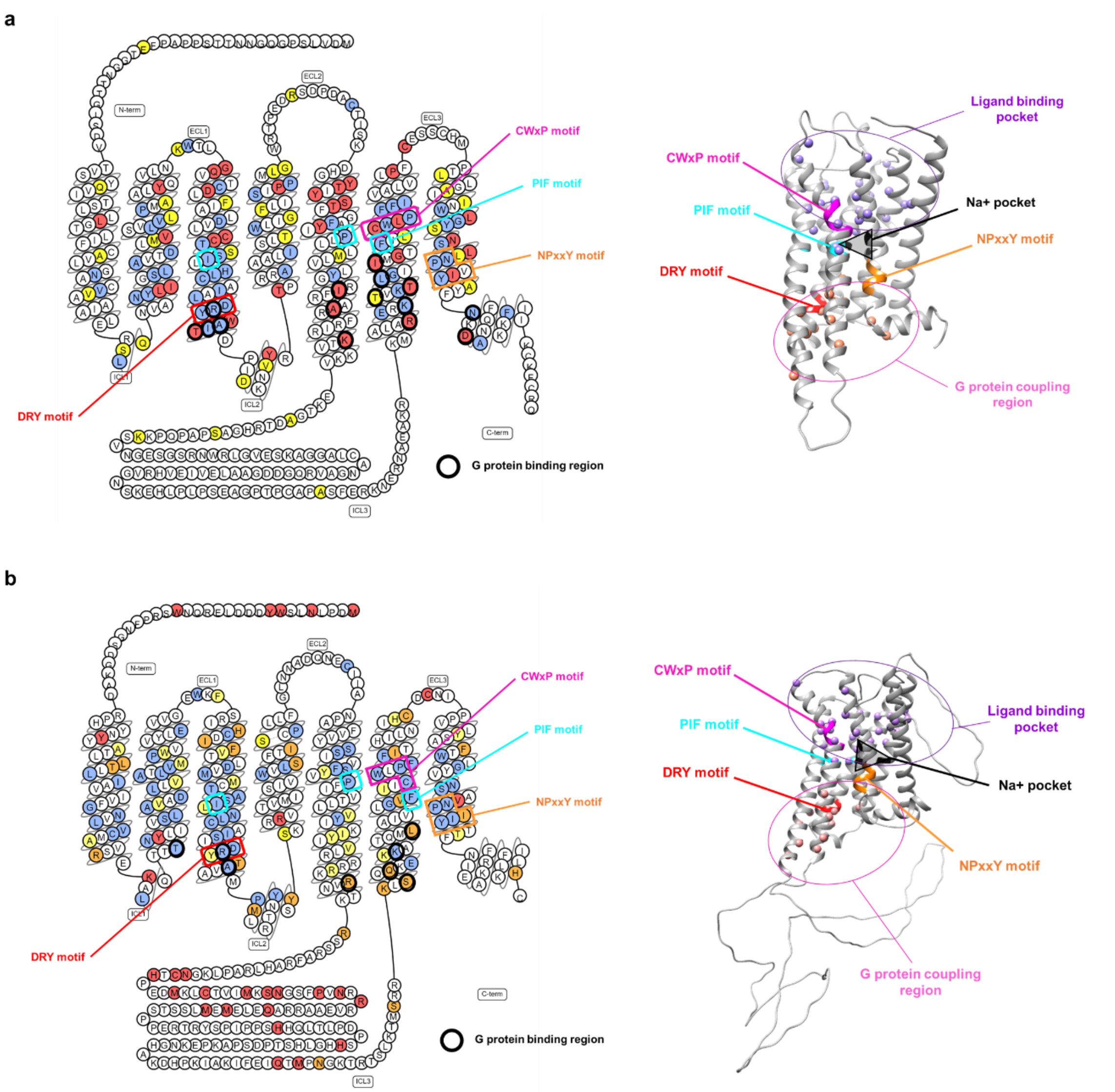

Supplementary figure S2. Details of HTR sectors and DAR sectors (a) Snake-like diagram and structure of HTR. (representing in modeling structure of active 5HT1A from GPCRdb) (b) Snake-like diagram and structure of DAR. (representing in modeling structure of DRD2 used in Fig. 3d) Each residue is colored by the color of sector name. At (b), orange color represents DAR-Orange1 and yellow represents DAR-Orange2. Among the sector residues, G protein binding regions are marked with a black thick border. In the structure representations, residues related to ligand binding and G protein coupling are shown only if they are involved in sector positions.

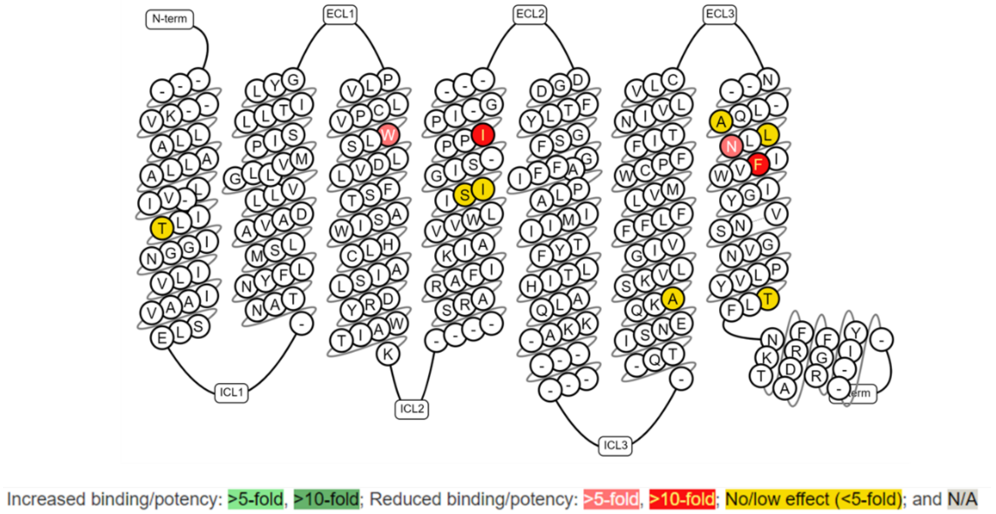

	5HT1A	5HT1B	5HT1D	5HT2A	5HT2B	5HT2C	5HT4R	5HT6R	5HT7R
**1.46**	< 5-fold								
**34.54**	< 5-fold			< 5-fold					
**3.28**							>10-fold	>5-fold	
**4.53**							< 5-fold	<5-fold	
**4.54**							< 5-fold		
**4.61**	< 5-fold	< 5-fold							
**6x33**	< 5-fold							< 5-fold	
**7x32**									<5-fold
**7x35**		< 5-fold		>10-fold	>10-fold	< 5-fold			<5-fold
**7x37**				>5-fold					
**7x43**	< 5-fold								
**7x54**			< 5-fold						

Supplementary figure S3. Mutant effects to ligand binding of HTR-Yellow. Increasing or reducing patterns are different for each position and subtypes. Coloring represents the increased binding/potency (>5-fold, >10-fold), reduced binding/potency (>5-folds, >10-folds) or No/low effects(<5-fold).

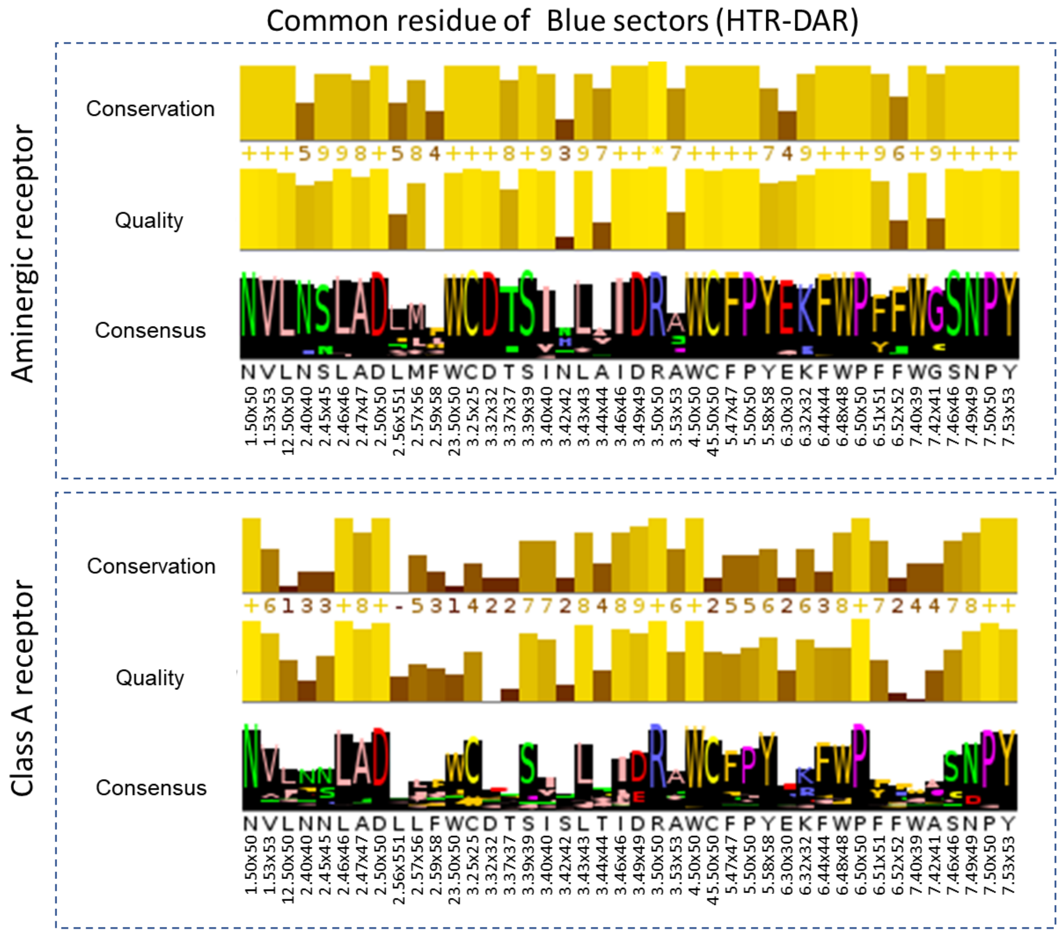

Supplementary figure S4. Common conserved residues of HTR-DAR blue sectors. The common residues were also conserved in aminergic receptor families (upper panel). Some residues changes to low conserved state in class A receptor family while the other residues showed high conservation level. (lower panel) ‘Conservation’ is physico-chemical property conserved for each position, ‘Quality’ is the likelihood of observing the mutations, and ‘Consensus’ is the percentage of the model residue per columns. Plots were generated by Jalview.[2]

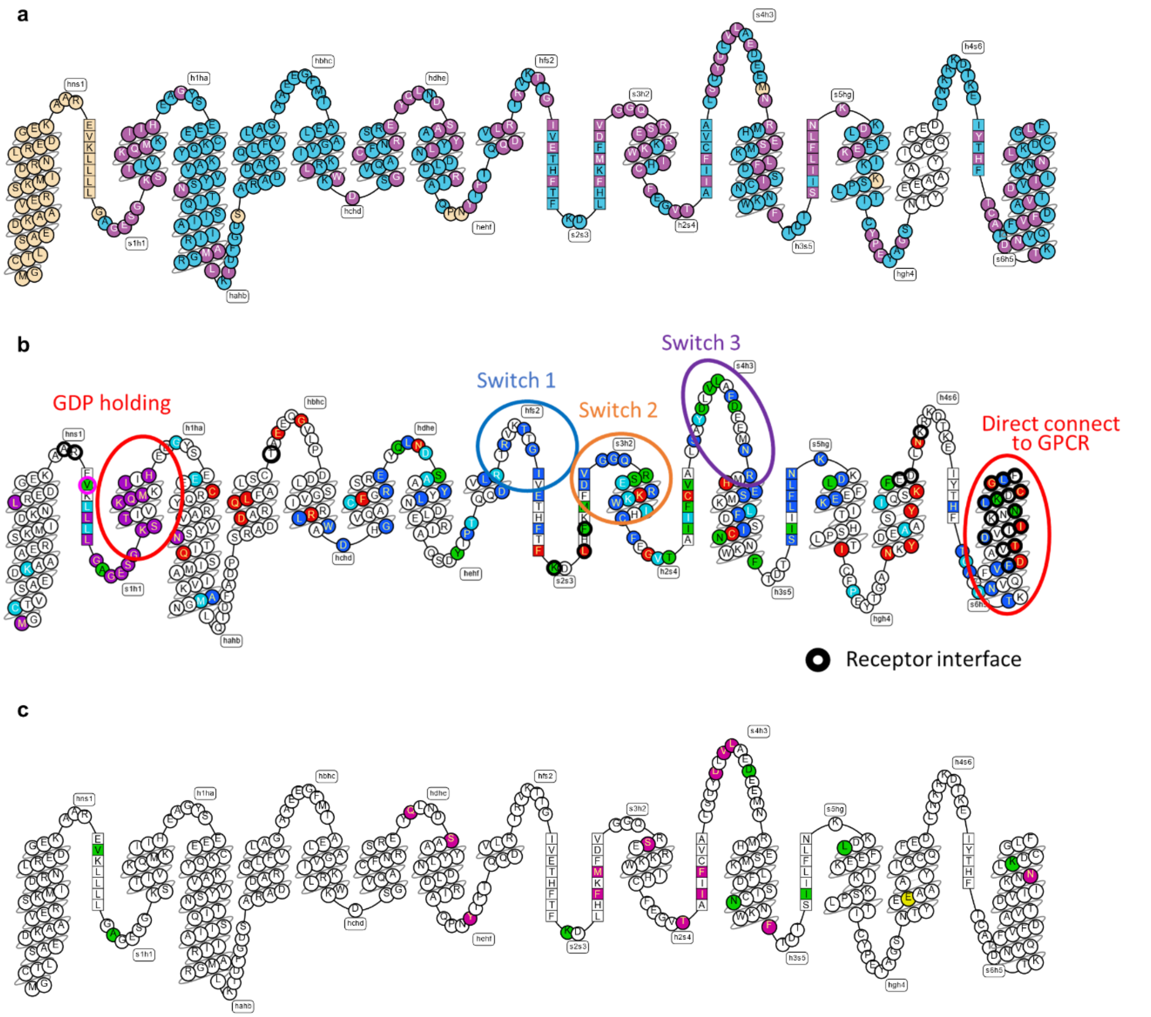

Supplementary figure S5. Details of G protein sectors. (a) Residue classification by evolutionary concepts defined by *Flock et al*.[3] Purple (conserved), Blue (selectivity barcode), Yellow (evolutionary neutral), and White (paralogue-specific). (b) G protein sector with color representation. (G-Red: red, G-Green: green, G-Blue1: cyan, G-Blue2: purple, G-Blue3: blue) (c) Further analysis of G-Green depending on overlapping with the selectivity barcode (Green) and conserved residues (Purple). Yellow residue is neither the selectivity barcode nor the conserved residues.

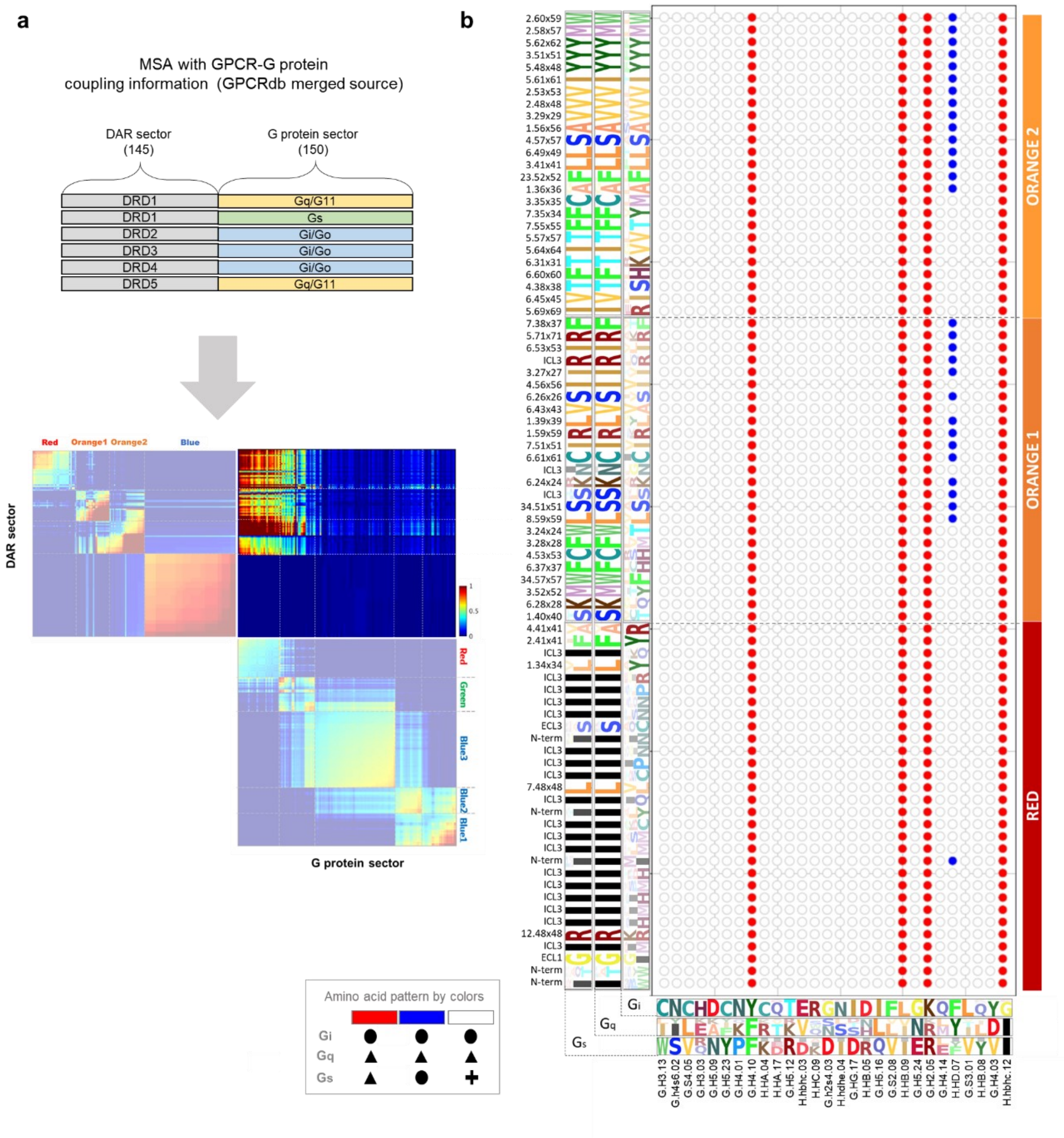

Supplementary figure S6. Results of integrated SCA of DAR and pattern analysis between DAR coevolved sector and G protein red sector. (a) MSA construction process with coupling information and the results of integrated SCA of DAR. DAR-Red and G-Red cross sections have high coevolution value. In addition, G-Red is also coevolving with DAR-Orange1 and DAR-Orange2. (b) Pattern analysis results of DAR-Red, Orange1, Orange2 and G-Red. Most of the pairs are having different amino acid distribution depending on G protein subtypes. Patterns of each color are explained in legend. Same shape in each column represents same amino acids. On sequence logo, black bar means 'Gap position' in MSA.

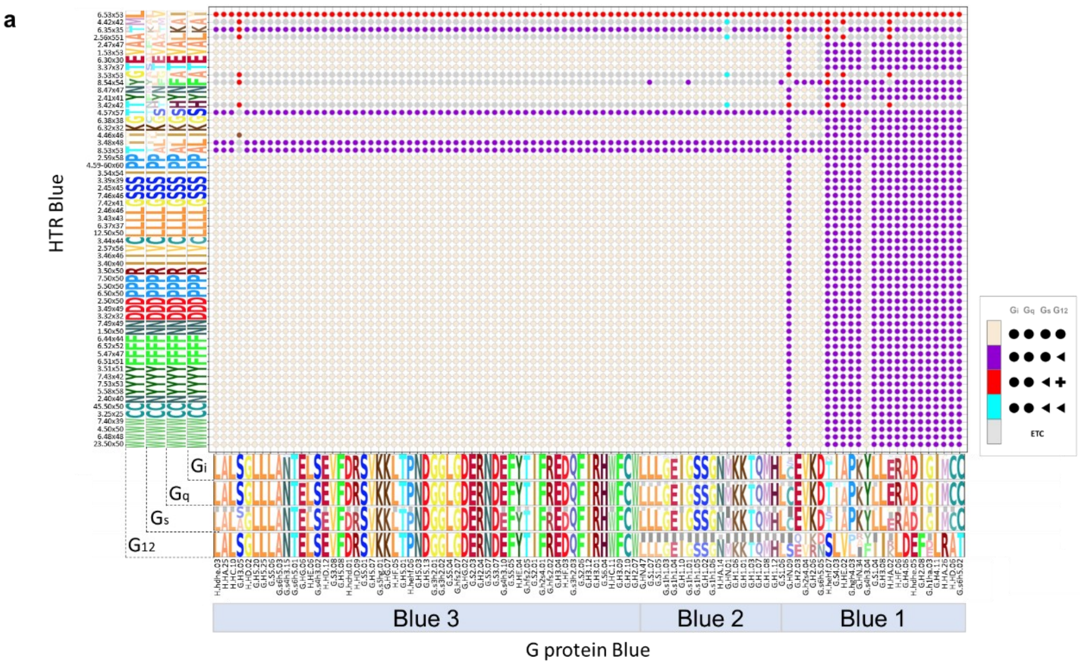

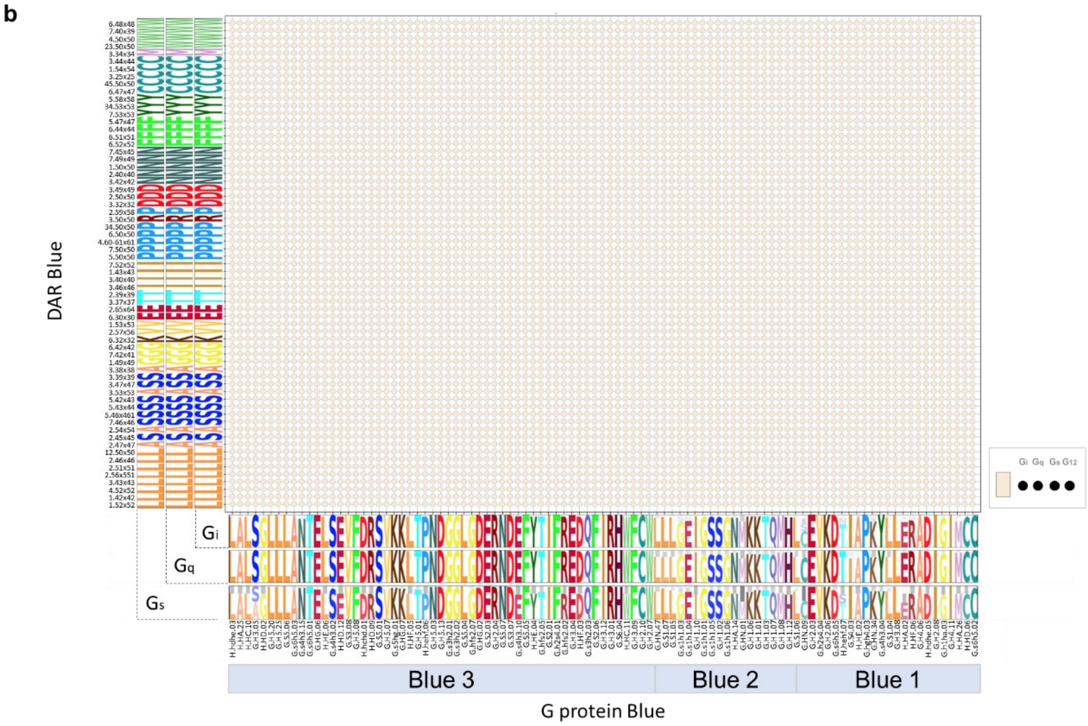

Supplementary figure S7. Pattern analysis between (A)HTR (B)DAR blue sector and G protein blue sector. (A) Pattern analysis results of HTR-Blue and G-Blue. Most of the pairs are having same patterns regardless of G protein subtypes, but in case of G-Blue1 residues, they are distinct by G12 subtype. (B) Pattern analysis results of DAR Blue and G-Blue. All of the pair between DAR-Blue and G-Blue are having same amino acid patterns.

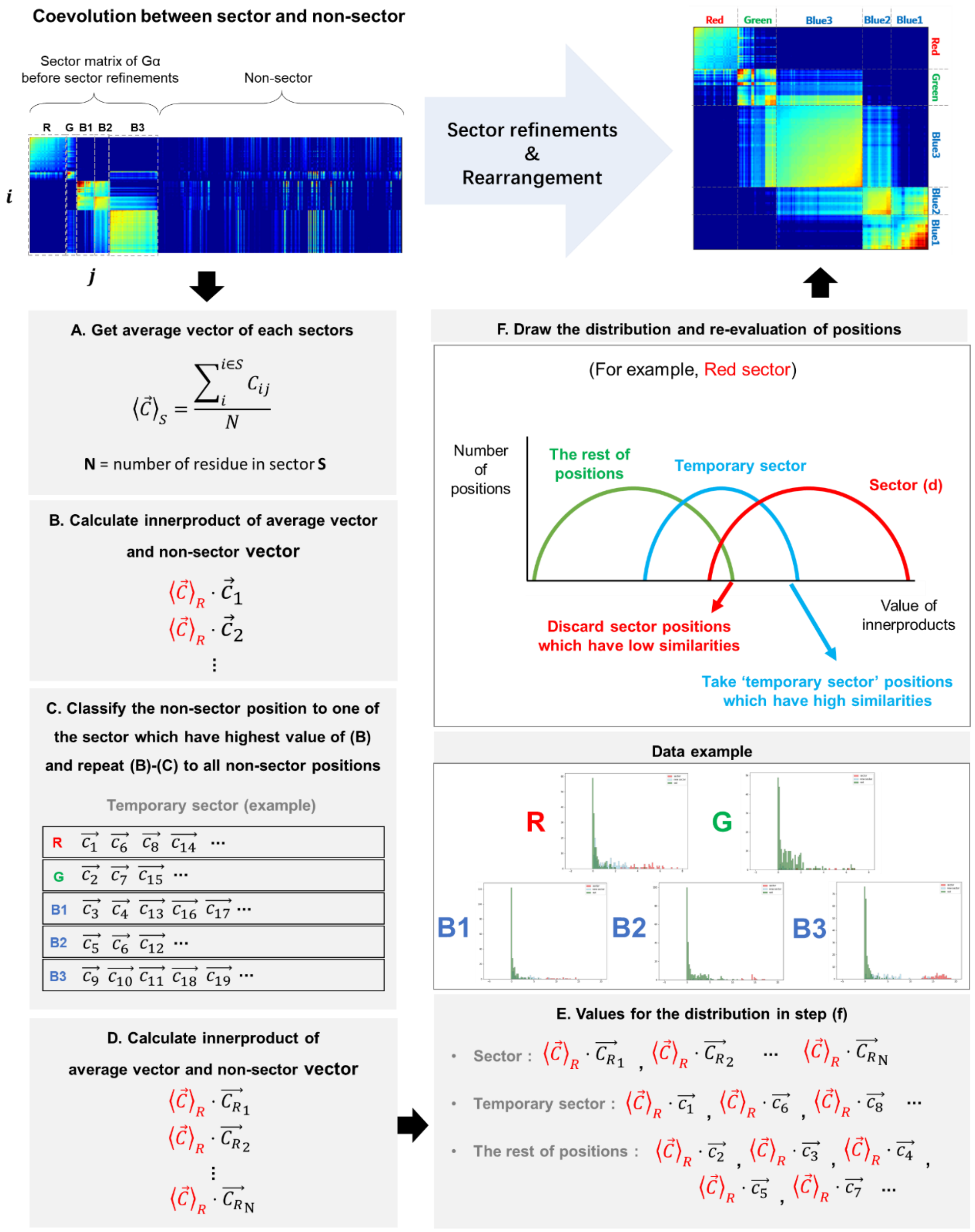

Supplementary figure S8. Flow scheme of Sector refinements. Sector refinements steps are described in order. Basic principle is taking positions with high similarities in non-sector, and discarding positions with low similarities in sector. We explain with G-Red sector for an example, however, the distribution of innerproducts can be differ from case to case.

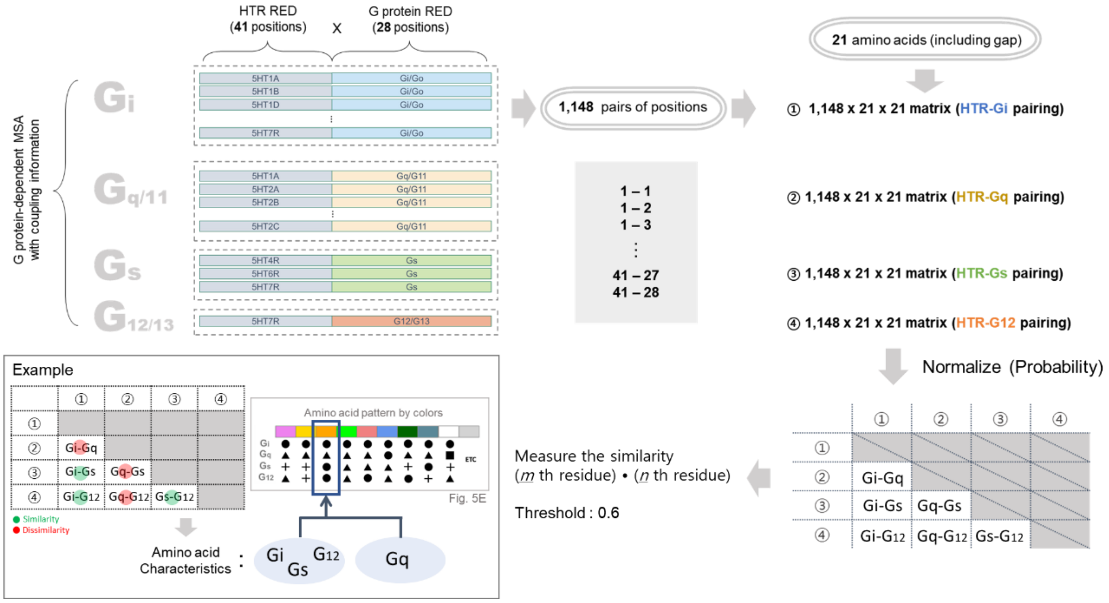

Supplementary figure S9. Example flow scheme of pattern analysis between HTR-Red and G-Red. The box at left below is one example of how we classified the pair of residues while pattern analysis. The whole results are representing in Fig. 5e, Supplementary Fig. S4b, S5a and S5b.

Supplementary text - Details of methods

Multiple sequence alignment (MSA)
Multiple sequence alignments (MSA) we used for this study were gathered from GPCRdb(https://gpcrdb.org) database. On GPCRdb, receptor sequence alignments tab, there are two options – sources and species. We selected 'Swiss-Prot' and 'All species' to build MSA of GPCRs. For 5-HT receptor family, the alignment consisted of 75 sequences and 955 positions. For dopamine receptor, the alignment consisted of 31 sequences and 629 positions. Large amount sequences were usually recommended for coevolution analysis, however, we only used sequences that skewed to human species. When calculating with small data sets, the observed frequency could be zero and it occurs zero-frequency problem. Therefore, we added a row of gaps into the alignments to avoid zero-frequency problem and it provided zero-offsets effects which is adding a constant. For G protein, we selected 'All sources' and 'All species', and the alignments consisted 325 sequences and 429 positions.

Statistical coupling analysis (SCA)
Statistical coupling analysis (SCA) is based on multiple sequence alignments (MSA) of a target protein across species. $D_i^{(a)}$ is conservation level of and amino acid *a* at position *i* in MSA. $f_i^{(a)}$ is relative entropy of the frequency of *a* at position *i*. Background frequency of *a* is represented as $q(a)$. The relative entropy of the prevalent amino acid *a* at each position is represented as (Eq.1),

$$D_i^{(a)} = f_i^{(a)} \ln \frac{f_i^{(a)}}{q(a)} + \left(1 - f_i^{(a)}\right) \ln \frac{1-f_i^{(a)}}{1-q(a)}. \quad \text{(Eq.1)}$$

and overall conservation at each position are plotted below. Top panels are the conservation of each positions *i* computed by relative entropy in the target protein. Bottoms are histogram of its relative entropy. (a) HTR, (b) DAR, and (c) G protein

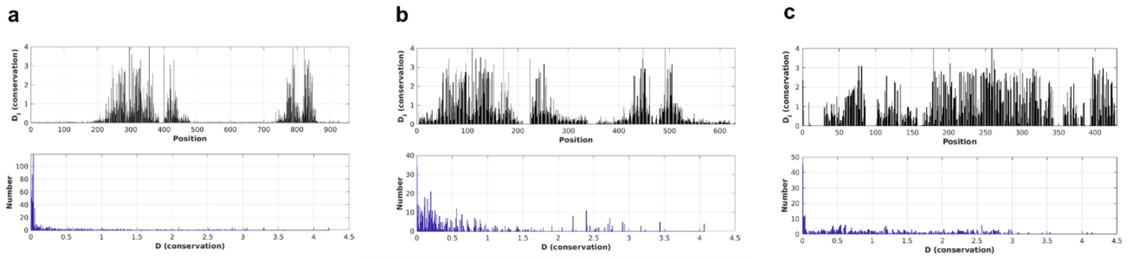

SCA correlation matrix is frequency-based correlation and weighted by positional conservation values. (Eq. 2).

$$\tilde{C}_{ij}^{ab} = \phi\left(D_i^{(a)}\right)\phi\left(D_j^{(b)}\right)C_{ij}^{ab} \quad \text{(Eq.2)}$$

Overall, position-by-position correlation matrix for our target proteins was constructed: (a) HTR, (b) DAR, and (c) G protein.

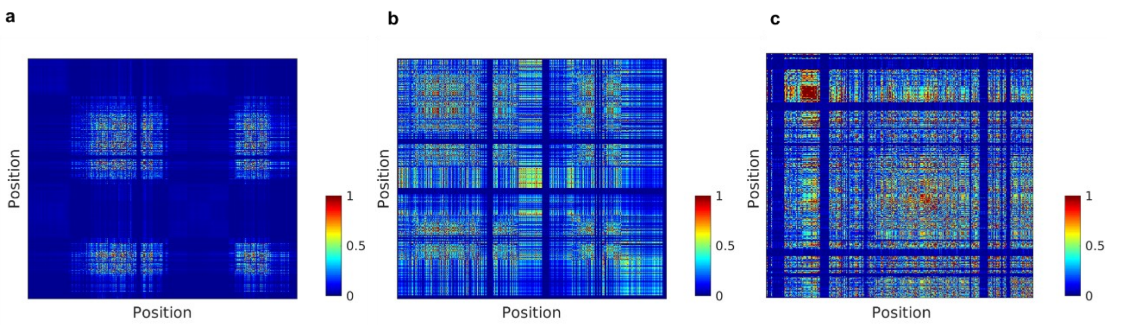

The SCA 5.0 toolbox used for SCA was implemented in MATLAB (The MathWorks, Inc., Natick, MA, USA). The specific details of mathematical procedures are described in previous literatures.[4]

Noise cleaning and sector identification

Position-by-position matrix is needed to do noise cleaning for sector identification. According to *Halabi et al*[4], two different noise could be occurred during SCA, statistical noise and historical noise which are not functionally significant. To remove historical noise, first eigenmode was not under consideration to exclude global correlation of phylogenetic relationship between sequences.[5] Statistical noise could arise because of limited sampling and it can be deduced by comparison to randomized sequences. For additional confirmation, we checked matrices of each eigenmode and reflected eigenmodes which containing significant signals for noise cleaning. In case of HTR, eigenmode 2 to 7 were interpreted, and eigenmode 2 to 5 for DAR. In case of G protein, eigenmode 2 to 3 were interpreted for sector identification.

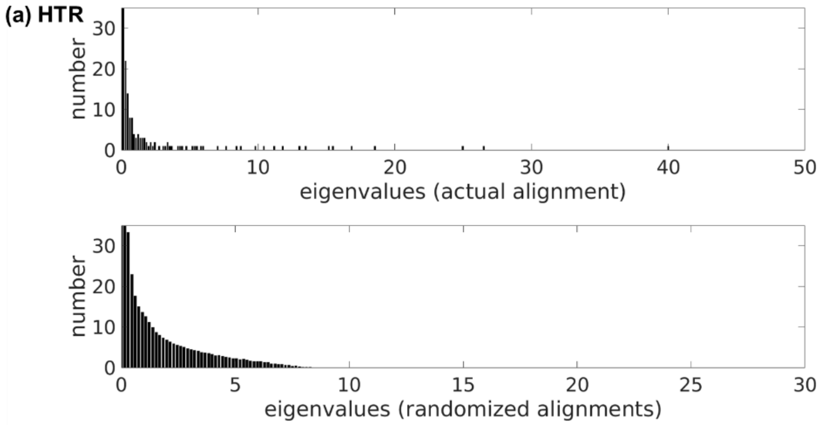

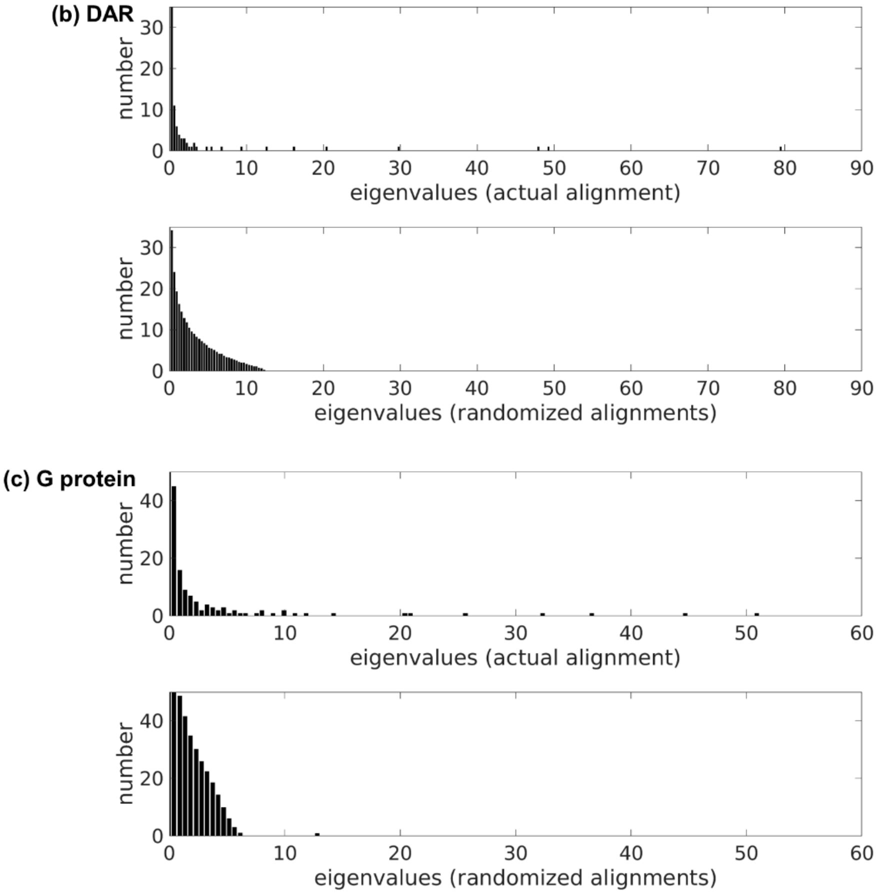

Sector identifications are based on visualized eigenvector of each residues and demarcated notable clusters of residues. The significance threshold was 0.05. After initial identification of sectors, we designed an algorithm to filter out the possible significant correlation we might miss during the processes and called as 'vector analysis'. It is based on a vector similarity comparison. Details are described in (Supplementary Fig.S8) and the results were plotted below. Vector analysis was effective in the final outcomes of HTR, DAR and G protein.

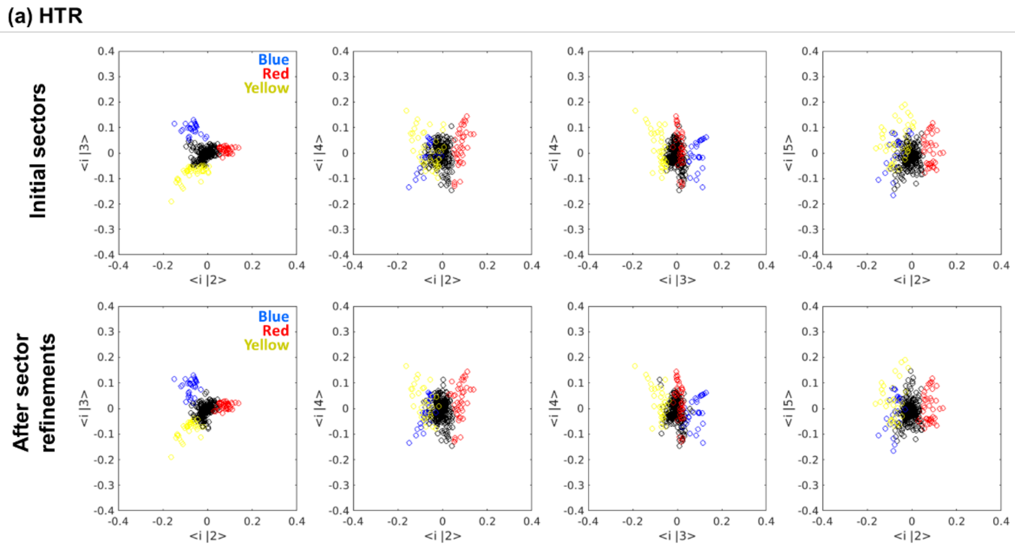

(b) DAR

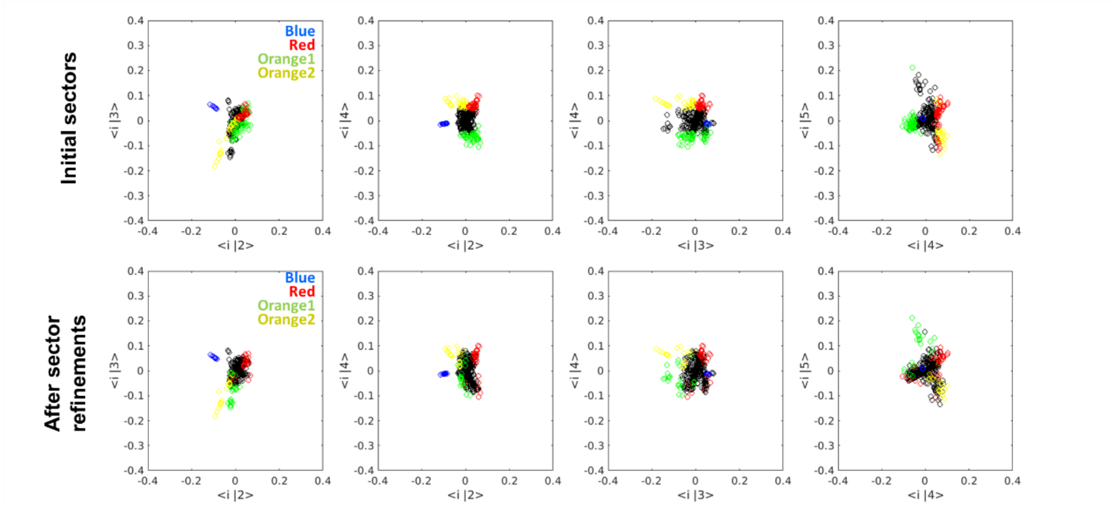

(c) G protein

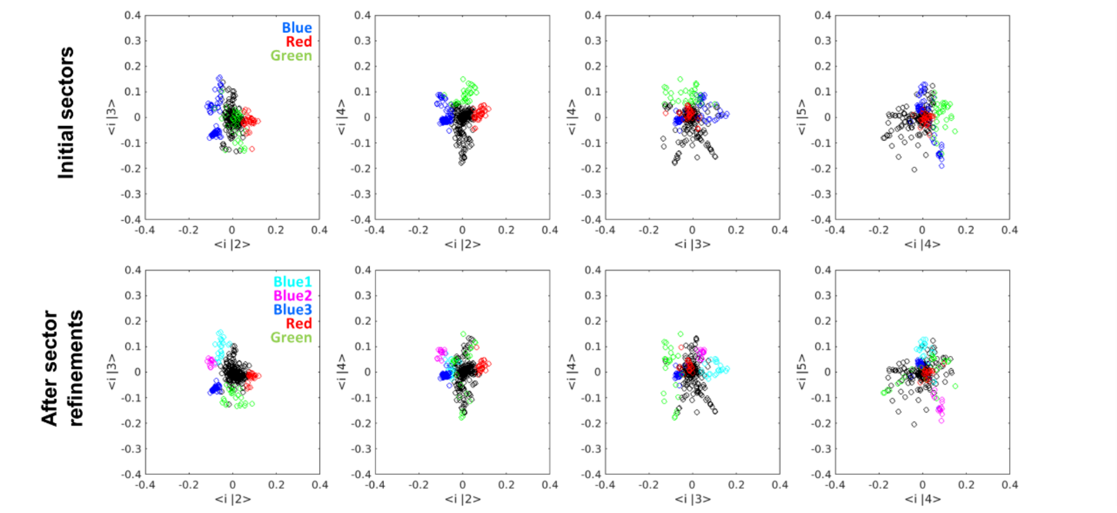

GPCR-G protein integrated SCA

To investigate coevolution between GPCR and G protein and how it is related to selectivity mechanisms, we built new joint alignments. We only used the sector residues for joint alignments because it is the outcomes that already filtered from coevolutionary analysis. The coupling information between GPCR and G proteins are also reflected in joint alignments. For example, the number of sector position of HTR family is 132. For G protein, 150 positions are in the sector. Therefore, we combined them and generated an integrated alignment with 282 positions. HTR1A coupled with Gi/o and Gq/o subtype of G proteins. Therefore, we doubled the HTR1A sequences and concatenated it to the sector residues of Gi/o and Gq/o respectively. In the case of HTR1B, it coupled to Gi/o subtype, so we concatenated the sector residues of HTR1B to the sector residues of Gi/o protein. We repeated this process to whole HTR, DAR subfamilies. The coupling information were gathered from GPCRdb, especially the merged one from two different sources (Guide to Pharmacology and *Inoue et al*[1]) and only primary coupling.

Integrated SCA was conducted by using the joint alignment described above. To analyze the correlation between two protein, we checked position-by-position matrix which is before the noise cleaning and sector identification steps.

GPCR-G protein pattern analysis

The significant correlations between GPCR and G protein were observed, then we tried to define coevolutionary patterns underlying the selectivity mechanisms. We observed every combination of sectors between GPCR and G protein. Among them, one case that showed significant correlations are represented in Fig. 5d, 5e and let us explained the analysis process using this case as an example. (Supplementary Fig. S9)

Fig. 5d and 5e are representing the pattern between HTR-Red and G-Red. We rebuild the joint alignment of HTR-G protein into four alignments according to G alpha subtypes (HTR-Gi, HTR-Gq, HTR-Gs, HTR-G12). The alignment was involved only sector residues of HTR-Red (41 positions) and G-Red (28 positions). We generate four set of matrices for 1,148 pair of HTR-Red and G-Red (41×28 pairs) and counted 21×21 amino acid frequencies (20 amino acid plus gap). Amino acid frequencies of the pairs are representing in Fig. 5d. We normalize the matrices to probabilities and measure the similarities between G protein subtypes. Then, we assort them into different pattern with color-coding at Fig. 5E. Threshold for similarity decision was 0.6.

Statistical validation of GPCR-G protein integrated SCA

We applied randomized process to validate of GPCR-G protein coevolution. Especially, we tested it for two sets: (1) HTR-Red and G-Red (upper panels), (2) DAR-Red, Orange1,2 and G-Red (lower panels). We shuffled the sequences of sector residues and remeasured the correlation between the GPCR-G protein for 10000 trials. Then, we extracted the maximum values (upper middle and lower middle panels) and calculated the mean values (upper right and lower right panels) of each pair for 10000 trials. As the final outcomes, the highly coevolved signals between GPCRs and G protein were not repeated in random trials, which suggested GPCR-G protein coevolution is statistically meaningful.

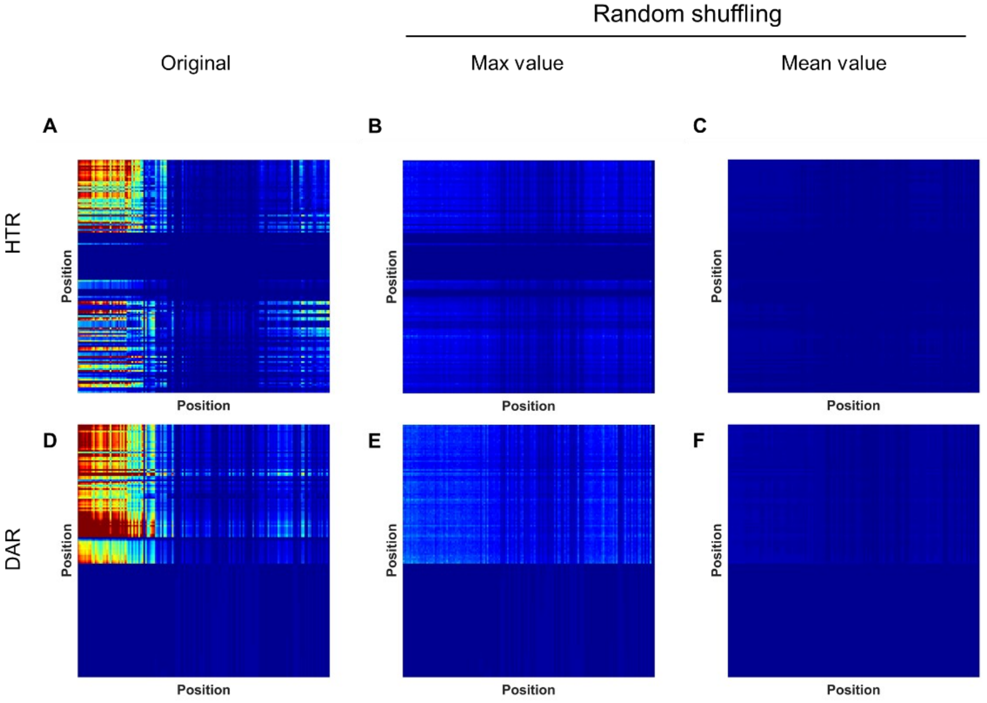

Supplementary dataset S1 (separate file). Subtype Classification by sectors

Supplementary dataset S2 (separate file). Ligand-sector interaction of crystal structures

References

1. Inoue, A. *et al.* Illuminating G-protein-coupling selectivity of GPCRs. *Cell* **177**, 1933–1947 (2019).
2. Waterhouse, A. M., Procter, J. B., Martin, D. M. A., Clamp, M. & Barton, G. J. Jalview Version 2—a multiple sequence alignment editor and analysis workbench. *Bioinformatics* **25**, 1189–1191 (2009).
3. Flock, T. *et al.* Selectivity determinants of GPCR–G-protein binding. *Nature* **545**, 317–322 (2017).
4. Halabi, N., Rivoire, O., Leibler, S. & Ranganathan, R. Protein Sectors: Evolutionary Units of Three-Dimensional Structure. *Cell* **138**, 774–786 (2009).
5. Teşileanu, T., Colwell, L. J. & Leibler, S. Protein sectors: statistical coupling analysis versus conservation. *PLoS Comput Biol* **11**, e1004091 (2015).
